# Accurate binning of metagenomic contigs via automated clustering sequences using information of genomic signatures and marker genes

**DOI:** 10.1038/srep24175

**Published:** 2016-04-12

**Authors:** Hsin-Hung Lin, Yu-Chieh Liao

**Affiliations:** 1 Institute of Population Health Sciences, National Health Research Institutes, Miaoli County 35053, Taiwan

## Abstract

Metagenomics, the application of shotgun sequencing, facilitates the reconstruction of the genomes of individual species from natural environments. A major challenge in the genome recovery domain is to agglomerate or ‘bin’ sequences assembled from metagenomic reads into individual groups. Metagenomic binning without consideration of reference sequences enables the comprehensive discovery of new microbial organisms and aids in the microbial genome reconstruction process. Here we present MyCC, an automated binning tool that combines genomic signatures, marker genes and optional contig coverages within one or multiple samples, in order to visualize the metagenomes and to identify the reconstructed genomic fragments. We demonstrate the superior performance of MyCC compared to other binning tools including CONCOCT, GroopM, MaxBin and MetaBAT on both synthetic and real human gut communities with a small sample size (one to 11 samples), as well as on a large metagenome dataset (over 250 samples). Moreover, we demonstrate the visualization of metagenomes in MyCC to aid in the reconstruction of genomes from distinct bins. MyCC is freely available at http://sourceforge.net/projects/sb2nhri/files/MyCC/.

High-throughput shotgun sequencing is a powerful means to study genomics of microbial communities. It has been used to recover microbial genomes directly from environmental samples, *e.g.*, cow rumen^1^, human stool^2^, permafrost^3^, and surface seawater^4^. Although the assembly of metagenomes poses more complex and varied challenges than single-genome assembly, several assemblers have been developed that are specific for metagenomes, such as Meta-IDBA^5^, MetaVelvet^6^ and Ray Meta^7^.

With advances in sequencing technology, cost-effective deep sequencing of metagenomes provides the sequencing depth necessary for metagenome assembly. However, the binning of assembled contigs into species- or strain-level clusters remains a significant challenge. A number of approaches have been developed to bin metagenomic sequences using genomic signatures^8^,^9^,^10^,^11^, coverage profiles across multiple samples^2^,^12^,^13^, or a combination of the two techniques^14^,^15^,^16^. Emergent self-organizing maps (ESOM) have been used to cluster sequences by tetra-nucleotide frequencies^8^ or by time-series abundance profiles^13^, however, the definition of contour boundaries on the ESOM-based plots represents a laborious and cumbersome task. VizBin provides reference-independent visualization of metagenomes, but it also requires subsequent human-augmented binning^10^. MaxBin, an automated tool for metagenomic binning mainly based on tetra-nucleotide frequencies combined with one-sample coverage levels, was compared to ESOM in an attempt to demonstrate its automated nature and comparable performance^15^. CONCOCT and MetaBAT combine sequence composition and coverage across multiple samples to automatically cluster contigs into bins, however, both techniques require more samples (*e.g.* 50) to achieve better binning results^14^,^16^. Although CONCOCT and MaxBin perform automated binning and evaluate cluster completeness on the basis of marker genes, they do not provide further evidence of confidence in distinguishing a bin from others to prioritize binning sequences. An ideal binning tool should enable clear distinction of clusters (the visualization of metagenomic data) and automatically produce accurate binning results.

In this study, we developed MyCC to automatically bin metagenomic contigs based on genomic signatures (and additional coverage profiles) and to visualize the binning of such metagenomes. We demonstrate that MyCC not only outperformed CONCOCT, MaxBin and MetaBAT in binning metagenomes derived from a small sample, but also performed well in complex metagenomic samples. Furthermore, the appropriate visualization of metagenomes in MyCC allows for reconstructing genomes of distinct clusters.

## Results

### MyCC implementation

**Figure 1:**
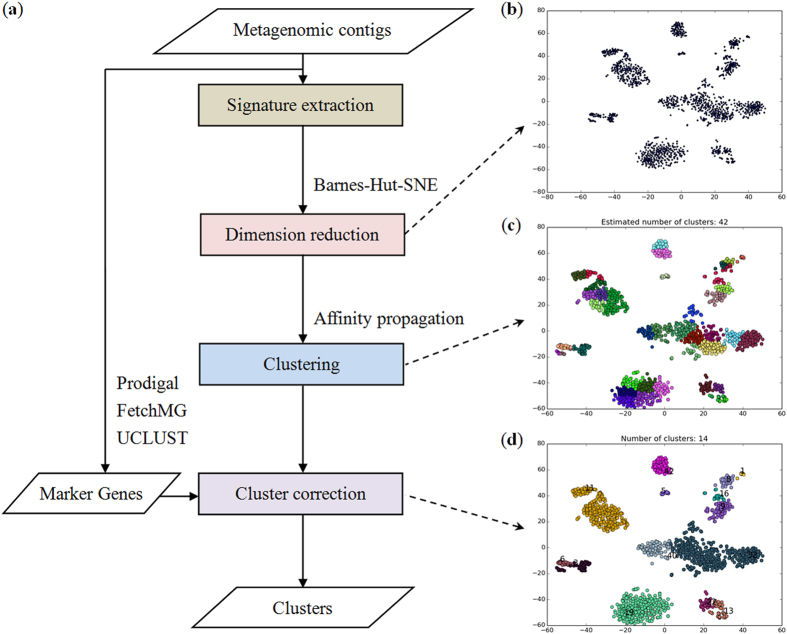
An overview of the MyCC workflow and visualization. (**a**) A schematic workflow for MyCC. (**b**) A plot of Barnes-Hut-SNE-based dimensionality reduction. (**c**) Automated clustering by affinity propagation. (**c**) Corrected clusters based on marker genes. These plots were output by MyCC in binning Sharon’s dataset (“MyCC.py carrol.fasta -a My.depth.txt -keep”).

MyCC was designed as an automated metagenomic binning tool, which allows binning of assembled metagenomic contigs without the need for reference sequences and manual intervention. We have developed MyCC as a virtual machine by deploying the required software including Prodigal^17^,^18^, FetchMG^19^,^20^, UCLUST^21^, BH-SNE^9^,^22^ and affinity propagation^23^ on Ubuntu Desktop 14.04.3 LTS; a schematic workflow of MyCC is shown in Fig. 1a. MyCC is open-source and available for download: (http://sourceforge.net/projects/sb2nhri/files/MyCC/). The detailed instruction for MyCC is also available at the link. Since MyCC was managed as a virtual machine, further software installations or configurations are not required. After importing the image file of MyCC, the user is able to bin metagenomic contigs. Genes on the metagenomes were predicted by Prodigal^17^ for the identification of a sequence that harbors single-copy marker genes using FetchMG^19^,^20^ along with UCLUST^21^. FetchMG extracts 40 universal phylogenetic marker genes^24^ by utilizing profile Hidden Markov Models trained on multiple sequence alignments of their orthologous groups that had been previously identified in prokaryotic genomes^20^, the 40 marker genes have been proven practical for the delineation of prokaryotic species^25^. For each contig, genomic signatures were obtained by calculating the count of occurrences for every kmer (*e.g.* 4 mer) and its reverse complement in that contig. After centered log-ratio (CLR) transformation^9^,^26^, the resulting high dimensional genomic signatures of metagenomic contigs were reduced to a two-dimensional scatter plot (Fig. 1b) using Barnes-Hut-SNE^22^. The scattered points corresponding to metagenomic contigs were clustered by affinity propagation (AP)^23^, as shown in Fig. 1c. The AP-generated clusters were finally corrected (Fig. 1d) based on the sequences harboring marker genes. For example, the six clusters (located in the lower-left side of Fig. 1c) are merged into one cluster (in Fig. 1d) because they are adjacent to each other and share marker genes. Due memory requirements for AP, a two-stage process was implemented for the binning of metagenomic contigs. The first stage was utilized to cluster relatively long sequences using the above-mentioned process, and the second stage was implemented to assign each of the remaining short sequences to a pre-defined cluster with a sequence possessing the minimum Euclidean distance between the 4 mer genomic signatures of the two sequences. As a default, MyCC processes a fraction of contigs (7/10) for first-stage clustering using 4 mer frequencies (-lt 0.7). As described in Supplementary Note, the command is as simple as “MyCC.py assembly.fa”.

### Recovering genomes from a metagenome community using MyCC

**Figure 2:**
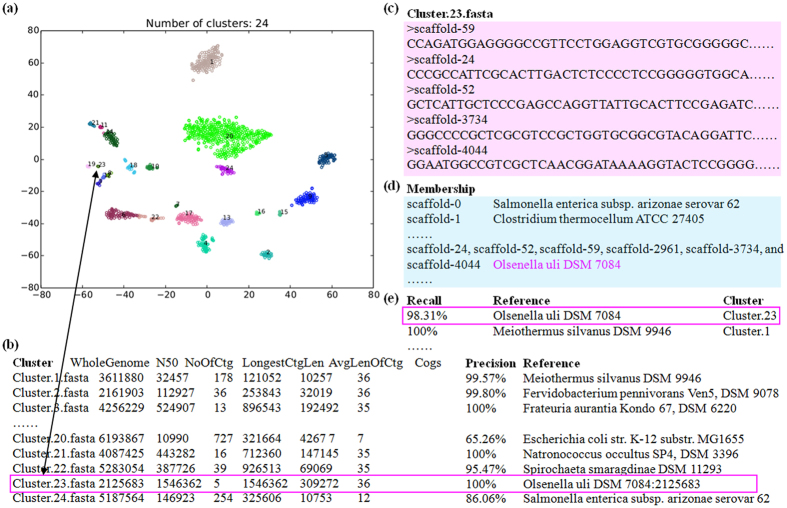
Explanations for outputs of MyCC. (**a**) Visualization of metagenomic binning. (**b**) A summary file produced by MyCC, reporting genome size (WholeGenome), N50, numbers of contigs (NoOfCtg) and marker genes (Cogs) for each bin. (**c**) Binning sequences in a cluster are output in FASTA format. (**d**) Gold-standard binning assignments available at MetaBAT’s website. (**e**) Binning performance evaluation based on the gold-standard assignments. MyCC was applied to bin a mock dataset of 25 genomes (“MyCC.py assembly.fa -a My.depth.txt”).

To demonstrate the effectiveness of MyCC, the software package was executed on a mock community available at MetaBAT’s website (https://bitbucket.org/berkeleylab/metabat)^16^. This community is composed of 25 known genomes. As illustrated by Fig. 2a,b, MyCC binned the metagenomic assembly along with the two-library coverage file (command: “MyCC.py assembly.fa -a depth.txt”) into the 24 clusters and produced a summary file to report genomic features including genome size (WholeGenome), N50, No. of contigs (NoOFCtg), and No. of marker genes (Cogs) for each cluster. Moreover, the metagenomic sequences binned by MyCC were organized into individual clusters. Here, we take Cluster.23 as an example. MyCC classified five centering contigs (in Fig. 2a) as Cluster.23 to constitute a 2.1 Mbp genome containing 36 marker genes (Fig. 2b) that is quite high for genome completeness. MyCC also generated a file named Cluster.23.fasta (Fig. 2c) to include the sequences of the five contigs that were binned into this cluster. According to the true assignment of each contig (Fig. 2d), we know that Cluster.23 corresponds to the genome of *Olsenella uli* DSM 7084. The recall for this genome (Fig. 2e) is 98.31% (2, 125, 683/2, 162, 161) and the precision of Cluster.23 is as high as 100% (shown in Fig. 2b). Overall, the binned contigs yielded high precision (95.87%) and recall (97.28%) for this mock community. Please note that the marker gene counts for Cluster.20 and Cluster.24 are low (7 and 12, respectively, as shown in Fig. 2b), suggesting that these two clusters are composed of fragmented contigs. In addition, owing to the genetic relatedness between *Escherichia* and *Salmonella*, MyCC was unable to distinguish them well and placed contigs of three species (*Escherichia coli* str. K-12 substr. MG1655, *Salmonella enterica* subsp. arizonae serovar 62 and *Salmonella bongori* NCTC 12419) into the two clusters (Cluster.20 and Cluster.24) (see Supplementary Note for details). Nevertheless, the vast majority of clusters produced by MyCC are complete and pure.

### Binning performance on various datasets

Two simulated metagenomes containing 10 and 100 bacterial species and two mock communities consisting of 25 and 64 genomes were used for evaluation of MyCC. Except for the mock dataset of 25 genomes provided by MetaBAT^16^, the other three datasets were designed and used for evaluation of metagenomic assembly^7^,^27^,^28^,^29^. The simulated reads for the 10 and 100 genomes and the sequencing reads of the 64-genome community were assembled separately into metagenomic conitgs by Ray Meta^7^. As displayed in Table 1, by simply inputting metagenomic contigs into MyCC, the sequences were clustered, by default, into 10, 93, 23 and 61 bins for the 10, 100, 25 and 64 metagenomes, respectively. It should be noted that CONCOCT^14^, MaxBin^15^,^30^ and MetaBAT^16^ all utilized the coverage information when binning the metagenomic contigs. Nevertheless, in the absence of coverage information, MyCC produced noteworthy binning performance based on its primary and secondary ratings in F1 scores (among the four tools). Comparing MyCC with CONCOCT, both packages assigned each contig (longer than 1,000 bp for CONCOCT, ≥1,000 bp for MyCC) to a bin. However, MyCC outperformed CONCOCT in terms of finding an accurate number of bins and higher F1 scores (89.0 vs. 74.0, 93.0 vs. 83.1 and 85.9 vs. 80.6 in the cases of 100, 25 and 64 genomes, respectively). MaxBin and MetaBAT produced an unclassified bin and unbinned some contigs, respectively, which resulted in high precision but compromised sensitivity (recall). For example, MetaBAT only binned 8,722 contigs out of the 23602-contig metagenomes (64 genomes) to yield a precision and recall of 86.78% and 77.40%, respectively. In addition to MyCC’s default settings, the noteworthy F1 scores were obtained by MyCC (highlighted in bold, Table 1) when combining 4 mer/5p6 mer frequencies with coverage information. Note that 5p6 mer represents a combination of penta-nucleotide (5 mer) and palindromic hexa-nucleotide (p6 mer). To provide a simple, real metagenomic dataset for the validation of MyCC, Sharon’s dataset was applied^13^. With the 18-run coverage profiles, MyCC successfully binned Sharon’s assembly into 14 bins with exceptional precision and recall (86.72% and 98.68%, respectively). Please also note that the five binning results of Sharon’s dataset were assessed by CheckM^31^ to provide estimates of genome quality (as shown in Supplementary Table S1). In agreement with the high precision and recall, MyCC was estimated to produce five high-quality genomes (completeness ≧95%, contamination ≦5% and strain heterogeneity ≦5%), but the other four binning tools produced only three. Accordingly, the superior performance of MyCC has been demonstrated in a direct comparison against other metagenomic binning tools (CONCOCT, MaxBin and MetaBAT) when applied to a small sample size.

### Applications of MyCC

Although most current metagenomic experiments encompass only a few samples, it has been reported that more complete genomes have been binned as the number of samples increased^14^,^16^. MyCC was applied to a benchmark dataset (MetaHIT dataset) provided by MetaBAT^16^. This dataset was derived from MetaHIT human gut metagenome data and contained 290 bacterial genomes. Along with the 264-run depth file provided for CONCOCT, MyCC binned the error-free metagenome contigs into 187 clusters. Among the 187 clusters, 96 clusters were characterized as possessing “good” binning performance (>90% precision and >50% recall). As evaluated on the MetaBAT website (https://bitbucket.org/berkeleylab/metabat/wiki/Home), this number (96 clusters) is substantially larger than those obtained by the other binning tools including Canopy, CONCOCT, GroopM^12^ and MaxBin (81, 56, 4, and 34 clusters, respectively), albeit it is the same as the one obtained by MetaBAT. In this fashion, MyCC was validated in its suitability to large-scale metagenomes. Furthermore, MyCC was applied to bin metagenomes of *Drosophila melanogaster* intestinal samples^32^. Sequencing reads of the *Drosophila* microbiota were *de novo* assembled by Ray Meta into 21,985 metagenomic contigs (≧1,000 bp). The contigs were then binned into 11 clusters by MyCC. Among the 11 clusters, three clusters with at least 75% of the marker genes were examined further for identification of the closest species. CheckM was also used to estimate the genome completeness and contamination of these clusters. The three clusters were assessed to be near complete (completeness ≧90%) with low contamination (≦5%). Finally, two new genomic sequences of *Acetobacter pasteurianus* and *Lactobacillus fructivorans* may be recovered from the clusters produced by MyCC (Cluster.5 and Cluster.6 in Supplementary Note). These two sequence files were separately annotated by Prokka^33^ to generate 2447 and 1055 coding sequences. In respect of number of coding sequences, the numbers of putative coding sequences are adequate because the protein numbers for *Acetobacter pasteurianus* IFO 3283-01 (NCBI reference sequence: NC_013209.1) and *Lactobacillus fructivorans* KCTC 3543 (RefSeq assembly: GCF_000185465.1) are 2621 and 1283, respectively. By virtue of the reference-independent approach, MyCC has been demonstrated, on the gut microbiota in flies and in infants, as a favorable tool for automated metagenomic binning.

## Discussion

MyCC provides an automated method to recover genomes from metagenomic assemblies using genomic signature information and single-copy marker genes. In the current work, MyCC was identified as the most optimal binning and visualization tool when applied to small sample sizes and was more than capable of binning large-scale metagenomes.

### Visualization of metagenomes

The visualization of metagenomic data via Barnes-Hut-SNE was previously proposed by Laczny *et al.*^9^. The authors implemented a program known as *VizBin* to render the visualization for human-augmented binning of metagenomic contigs^10^. In comparison with VizBin (Supplementary Fig. S1) on the Sharon’s dataset, MyCC not only provides the visualization of metagenomes with clearly separated clusters (Fig. 1b) but also performs automated clustering without reference genomes and *a priori* knowledge of the number of genomes (Fig. 1c,d); this feat is performed by incorporating coverage information and exploiting affinity propagation and along with single-copy marker genes. As illustrated in Fig. 1d, MyCC successfully organized *Enterococcus faecalis* contigs into the cluster (precision of 96.83%) found at the top-right corner (Cluster.1 with contigs in yellow color) to recover the genome of *Enterococcus faecalis* with a recall of 100%; however, it was difficult to locate the points of *Enterococcus faecalis* on the VizBin-produced plots (Supplementary Fig. S1). The scatter plot visualization combined with the summary file (illustrating genome size and number of marker genes for each cluster) in MyCC enable us to recover individual genomes from the metagenomes (as evident from the Fig. 2). Similar to cluster evaluation with single-copy marker genes in MaxBin^15^ and CONCOCT^14^, CheckM^31^ can be used after metagenomic binning to prioritize genome bins for post-binning processes^16^. A visualization plot and marker gene counts provided by MyCC have already addressed this need. We have demonstrated that the two draft genomes of *Acetobacter pasteurianus* and *Lactobacillus fructivorans* were recovered from Drosophila intestinal samples (Supplementary Note). Furthermore, the adoption of multiple parameter settings (*e.g.*, 4 mer/5 mer/5p6 mer, w/wo coverage information and one/two stages) substantiate MyCC’s versatility for various datasets; results are provided in Table 1.

### Parameter settings in MyCC

MyCC was applied to various metagenome datasets to systematically explore the effect of different settings, which include genomic signature (4 mer or 5p6 mer), one or two stages, and with or without coverage information; results are provided in Supplementary Fig. S2. Except for the simple community (10 genomes) that possessed a narrow coverage distribution (70-130X), incorporating coverage information with the genomic signature improves MyCC’s binning accuracy. Because it is unlikely to observe even coverage distribution in natural metagenomic communities, we would suggest including coverage information in MyCC, if available. In addition, we found that the signature setting on 4 mer and 5p6 mer sequences was advantageous for binning simple and complex metagenome communities, respectively. We therefore recommended that users leverage the default settings for genome number estimation, and subsequently select 5p6 mer when more than 50 clusters are produced. As for implementing MyCC in one or two stages, this matter depends on the computing system and influences computational efficiency. Due to memory requirements for affinity propagation, we have employed sparse similarity to AP and design two stages to partially address the need for extended system memory. All the datasets in Table 1 (23,602 conitgs at most) were able to be complete for binning by MyCC within 1.5 h using Intel Xeon E31245 CPU with 4 GB RAM (see Supplementary Note). In spite of this, future work should be directed at memory-efficient clustering as large datasets require large memory size.

### Comparison to automated binning tools

CONCOCT is an algorithm that combines sequence composition and coverage across multiple samples to automatically cluster contigs. It has been compared with LikelyBin, MetaWatt, CompostBin and SCIMM to demonstrate that it performs better than the four alternatives^14^. MaxBin uses one-sample coverage information in addition to tetra-nucleotides frequencies for automated binning^15^. MaxBin 2 (a new version of MaxBin) supports multiple samples at the same time, thereby enabling construction of multiple metagenomes^30^. MetaBAT serves as an efficient tool for reconstructing genomes from complex microbial communities; this is achieved by integrating probabilistic distances of genome abundance with sequence composition^16^. MyCC was compared to these three binning tools on datasets with small sample sizes (1 to 11 samples; note that Sharon’s dataset comprises 11 samples in 18 runs). GroopM required at least three samples for binning^12^, it was thus only applied to the Sharon’s dataset for comparison. The results given in Table 1 provide compelling evidence for accurate binning of MyCC on metagenomic contigs derived from a small size of samples. Relative performance of the various binning tools (evaluated by *benchmark. R*, available in MetaBAT’s website https://bitbucket.org/berkeleylab/metabat) on the five datasets can be found in Supplementary Fig. S3–S7. Those results are in line with Table 1 for verifying the applicability of MyCC. Although the execution time of MyCC for these datasets ranged from 10 minutes to 1.5 hours depending on the number of contigs, it is relatively shorter than the time required for metagenome assembling using Ray Meta (20 hours to 12 days). Parallel affinity propagation should be explored to accelerate the clustering process. In addition to the small samples, MyCC was applied to the MetaHIT dataset and the binning result was evaluated by *benchmark. R*. The binning performance of MyCC compared to Canopy, CONCOCT, GroopM, MaxBin and MetaBAT is displayed in Supplementary Fig. S8. MyCC exhibited improved recall in half of the bins at the cost of less precision; nevertheless, it nearly achieved the best F1 and F0.5 in the first-hundred bins. Based on the visualization of metagenomes (Fig. S9) in MyCC, separable and outer clusters with a moderate number of marker genes (*e.g.*, >25) can be selected for further investigation. For example, MyCC binned the metagenomic contigs into Cluster.4 with a precision of 97.71%, Cluster.7 with a precision of 99.35%, and Cluster.10 with a precision of 100% to recover the genome of *Alistipes putredinis* DSM 17216 with a recall of 83.93%, *Bacteroides pectinophilus* ATCC 43243 with a recall of 76.04%, and *Tannerella* sp. 6_1_58FAA_CT1 with a recall of 85.07%. Accordingly, applying MyCC to a metagenome community recovered genome sequences with a high degree of fidelity.

## Materials and Methods

### Implementation of MyCC

The MyCC algorithm was implemented in Python (https://www.python.org/). As illustrated in Fig. 1, Prodigal (v2.6.2) was applied for metagenomic gene prediction and translation^17^,^18^. FetchMG (v1.0), downloaded from http://www.bork.embl.de/software/mOTU/fetchMG.html, was subsequently employed to extract 40 single-copy universal marker genes^19^,^20^ from the predicted amino acid sequences. Sequences containing species-level marker genes were identified by UCLUST (v1.2.22q) with an identity threshold of 95%^21^. With respect to each contig, genomic signatures were obtained via calculation of the count of occurrences for every kmer and its reverse complement in that contig. In the case of tetra-nucleotides (4 mer), 136-dimensional genomic signatures of metagenomic contigs were produced. In addition to the 4 mer case, 5 mer (512 dimensions) and 5p6 mer (576 dimensions) features have been implemented in the signature extraction of penta-nucleotides and penta-nucleotides combined with palindromes of hexa-nucleotides, respectively. One pseudocount was added to eliminate zero counts; the counts were subsequently normalized by dividing by the sum of each contig signature. Subsequently, the normalized values for each signature were standardized via computation of the quotient between the signature and the geometric mean of that signature in a process referred to centered log-ratio (CLR) transformation^9^. In addition to the genomic signature, a coverage file was, as an option, provided to MyCC. In contrast to adding pseudocount to contig signature, only the non-zero depths were taken for CLR transformation. The processed high-dimensional genomic signatures (plus coverage information) of each contig was reduced to two dimensions by Barnes-Hut-SNE (v0.1.1) (https://github.com/danielfrg/tsne), which allows us to conveniently visualize the metagenomic contigs in a scatter plot (Fig. 1b)^22^. The following parameters were used for Barnes-Hut-SNE: perplexity of 20, theta of 0.5 and no PCA. The scatter points (representing contigs) were clustered by affinity propagation (Fig. 1c)^23^ with the following settings: maxits = 1000, convits = 15 and dampfact = 0.8. The executable software required to compute affinity propagation (apcluster_linux64) was downloaded from http://www.psi.toronto.edu/affinitypropagation/software/. Negative-squared Euclidean distances between pairs of data points were used as input measures of similarity for affinity propagation in order to cluster data. To perform affinity propagation efficiently, the squared Euclidean distances shorter than 500 were employed as the sparse similarity between two points (default: “-st 500”). The sequences containing species-level marker genes identified beforehand were employed for cluster correction. If sequences in a cluster were found to harbor more than two duplicate marker genes, the data points corresponding to that cluster were split into two clusters via a technique known as spectral clustering^34^. If sequences in two adjacent clusters were found to harbor complementary marker genes, the two clusters were merged into one. Such processes were iteratively performed until no cluster required segmentation or merging, results are shown in Fig. 1d. As a virtual machine, MyCC is fully automated and easy to use. It is also assembled as a docker container, which can run on a local host or in the Cloud.

### Simulated datasets

Simulated Illumina sequences for a low complexity (10 genome) metagenome were downloaded from http://www.bork.embl.de/~mende/simulated_data/27. A complex simulated metagenome (100 genomes) was produced with abundances following a power law by executing Ray Meta-associated scripts^7^. The two metagenome datasets (4 Gbp and 40 Gbp reads) were assembled *de novo* by Ray Meta (Ray version 2.3.1) with a k-mer length of 31 into 3,256 and 14,513 contigs, respectively. The reads were then mapped back onto the contigs to determine coverage with Bowtie 2^35^.

### Mock datasets

A metagenomic assembly of a mock community (25 genomes), along with two-library alignment files (.bam and .bai), were downloaded from http://portal.nersc.gov/dna/RD/Metagenome_RD/MetaBAT/Software/Mockup/16. Sequencing data (over 11 Gbp) of a mixture of archaeal and bacterial synthetic communities (64 genome), deposited in the NCBI Sequence Read Archive (SRA) under the Accession of SRR606249^29^, were downloaded for metagenome assembly with Ray Meta, resulting in 77,990 contigs. The reads were mapped to the contigs by Bowtie 2.

### Sharon’s dataset

An infant human gut microbiome has been analyzed for microbial genome reconstruction by Sharon *et al.*^13^. The authors produced a metagenome assembly (2,329 contigs) and provided the assembly along with binning information (carrol.scaffolds_to_bin.tsv) in http://ggkbase.berkeley.edu/carrol/. Sequence reads of 18 Illumina runs (SRR492065-66 and SRR492182-97) for the infant gut metagenome were downloaded from the NCBI SRA (SRA052203). After removing contigs shorter than 1,000 bp, the reads were mapped to 2,294 contigs with Bowtie 2 in order to produce coverage profiles of each run.

### *Drosophila* microbiota

A metagenomic approach has been taken to assess microbiota composition during *Drosophila* aging^32^. Sequencing data were downloaded from NCBI SRA (SRP061446) and assembled by Ray Meta. The sequencing reads were mapped to the Ray Meta-assembled contigs for producing coverage profiles. The metagenomic assembly and the coverage profile were input to MyCC for binning.

### Performance evaluation

The Ray Meta-assembled contigs for the 10-genome, 64-genome and 100-genome metagenomes were aligned against reference genomes using BLAST to define the gold-standard binning assignments. For the 25-genome and Sharon’s datasets, the binning assignments (as gold standards) were downloaded directly from their respective websites (http://portal.nersc.gov/dna/RD/Metagenome_RD/MetaBAT/Software/Mockup/ and http://ggkbase.berkeley.edu/carrol/, respectively). Given the availability of gold standards, we computed precision and recall to evaluate binning performance^14^,^15^. Assume there are N genomes in the dataset, which were binned into M clusters. The overall precision and recall are calculated as equations (1) and (2)


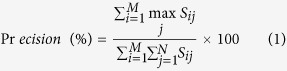



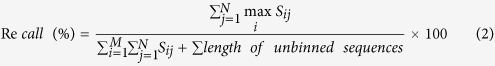


in which 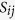 indicates the total length of contigs in a cluster *i* corresponding to a reference genome *j*. In addition to the precision and recall, an *F1* score (equation (3)) is used to weigh both measurements by taking their harmonic mean:


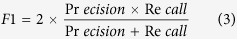


To benchmark MyCC against a synthetic metagenomic assembly obtained from 264 MetaHIT human gut metagenome data (the MetaHIT dataset^16^, available at https://bitbucket.org/berkeleylab/metabat/wiki/Home), the 195,601 contigs in the filtered assembly were binned by MyCC along with the depth file for CONCOCT (command: “MyCC.py assembly-filtered.fa -lt 0.4 -st 50 -a depth_concoct.txt 56 mer”). Additionally, the binning results of Canopy, CONCOCT, GroopM, MaxBin and MetaBAT (bin1, sensitive mode) were separately downloaded from the folder of results in http://portal.nersc.gov/dna/RD/Metagenome_RD/MetaBAT/Files/. These results were all evaluated by *benchmark. R* (provided in the link) to demonstrate binning performance (see Supplementary Methods).

## Additional Information

**How to cite this article**: Lin, H.-H. and Liao, Y.-C. Accurate binning of metagenomic contigs via automated clustering sequences using information of genomic signatures and marker genes. *Sci. Rep.* **6**, 24175; doi: 10.1038/srep24175 (2016).

## Supplementary Material

Supplementary Information

## Figures and Tables

**Table 1:** Binning performance on various datasets (simulated reads, mock libraries and real samples).

		No. of bins	No. of binned contigs	Precision (%)	Recall (%)	F1 (%)
Simulated dataset	**10 Genomes (2,185 contigs**^a^)					
	CONCOCT	19	2,185	98.78	97.67	98.2
	MaxBin	10	2,125	93.16	97.17	95.1
	MetaBAT	9	1,653	90.26	95.13	92.6
	MyCC (default)	10	2,185	97.79	97.79	97.8
	**MyCC (one stage)**	**11**	**2,185**	**99.17**	**98.45**	**98.8**
	**100 Genomes (8,978 contigs**^a^)					
	CONCOCT	79	8,977	59.67	97.40	74.0
	MaxBin	84	7,308	89.64	84.52	87.0
	MetaBAT	105	5,430	92.72	89.59	91.1
	MyCC (default)	93	8,978	87.45	90.54	89.0
	**MyCC (5p6 mer, cov)**	**88**	**8,978**	**89.68**	**94.09**	**91.8**
Mock datasets	**25 Genomes, 2 libraries (1,893 contigs**^a^)					
	CONCOCT	29	1,892	72.67	97.15	83.1
	MaxBin2	26	1,892	90.00	90.38	90.2
	MetaBAT	31	1,742	93.78	93.57	93.7
	MyCC (default)	23	1,893	88.97	97.35	93.0
	**MyCC (4 mer, cov)**	**24**	**1,893**	**95.87**	**97.28**	**96.6**
	**64 Genomes (23,602 contigs**^a^)					
	CONCOCT	84	23,585	70.63	93.90	80.6
	MaxBin	56	20,639	84.96	81.83	83.4
	MetaBAT	70	8,722	86.78	77.40	81.8
	MyCC (default)	61	23,602	83.19	88.76	85.9
	**MyCC (5p6 mer, cov)**	**57**	**23,602**	**84.36**	**92.85**	**88.4**
Real dataset	**Sharon’s dataset, 18 runs (2,294 contigs**^a^)					
	CONCOCT	32	2,291	79.92	97.58	87.9
	GroopM	13	1,687	88.39	86.29	87.3
	MaxBin2	10	2,294	82.94	93.75	88.0
	MetaBAT	10	1,573	85.46	93.66	89.4
	**MyCC (4 mer, cov)**	**14**	**2,294**	**86.72**	**98.68**	**92.3**

^a^Only contigs with a length longer or equal to 1,000 bp.
