# Supplementary material for: Accurate binning of metagenomic contigs via automated clustering sequences using information of genomic signatures and marker genes
Source: Sci Rep. 2016 Apr 12;6:24175. doi: 10.1038/srep24175 (PMC4828714; doi:10.1038/srep24175)
Supplement: Supplementary Information [file srep24175-s1.pdf]

Supplementary Information

Title: Accurate binning of metagenomic contigs via automated clustering sequences using information of genomic signatures and marker genes

Author list: Hsin-Hung Lin and Yu-Chieh Liao

Index

Figure S1 2

Figure S2 3

Figure S3 4

Figure S4 5

Figure S5 6

Figure S6 7

Figure S7 8

Figure S8 9

Figure S9 10

Table S1 11

Supplementary Note 13

Install MyCC 13

Run MyCC 16

10 Genomes 16

100 Genomes 23

25 Genomes 25

64 Genomes 28

Sharon’s Dataset 30

Drosophila Dataset 36

Docker of MyCC 44

Supplementary Methods 46

(a)

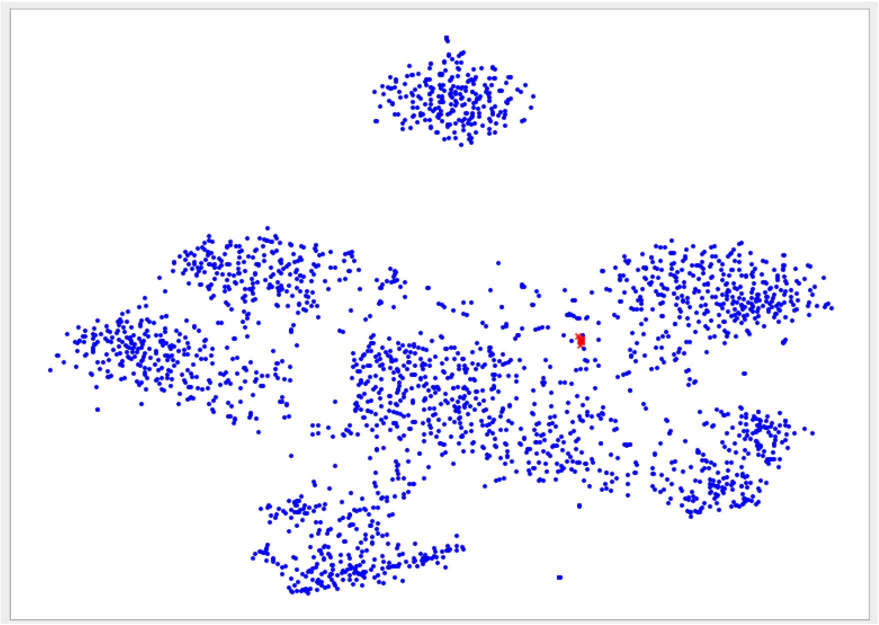

(b)

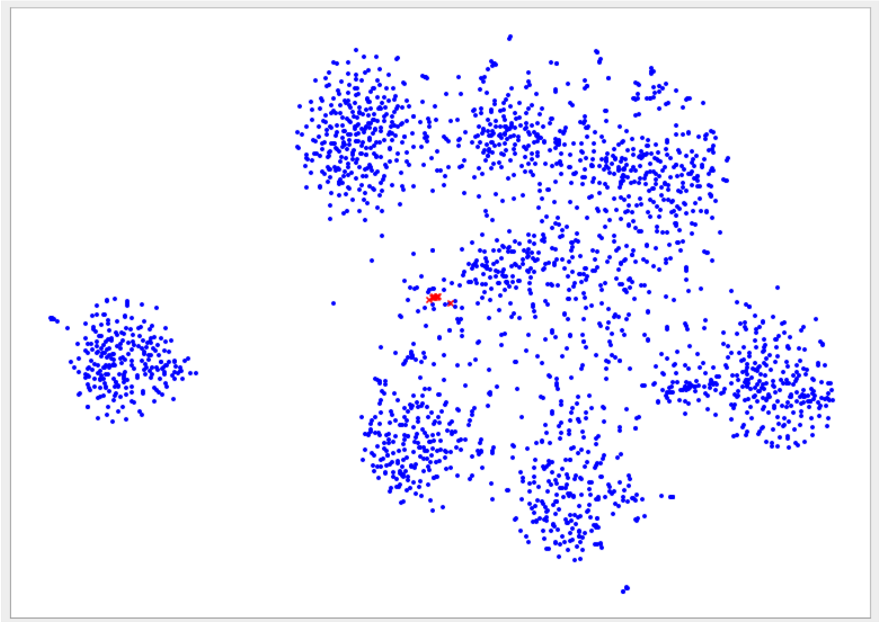

Figure S1 Scatter plot visualization in VizBin for Sharon's dataset employing: (a) 5mer signatures and (b) 4mer signatures. Red crosses highlight contigs of *Enterococcus faecalis*.

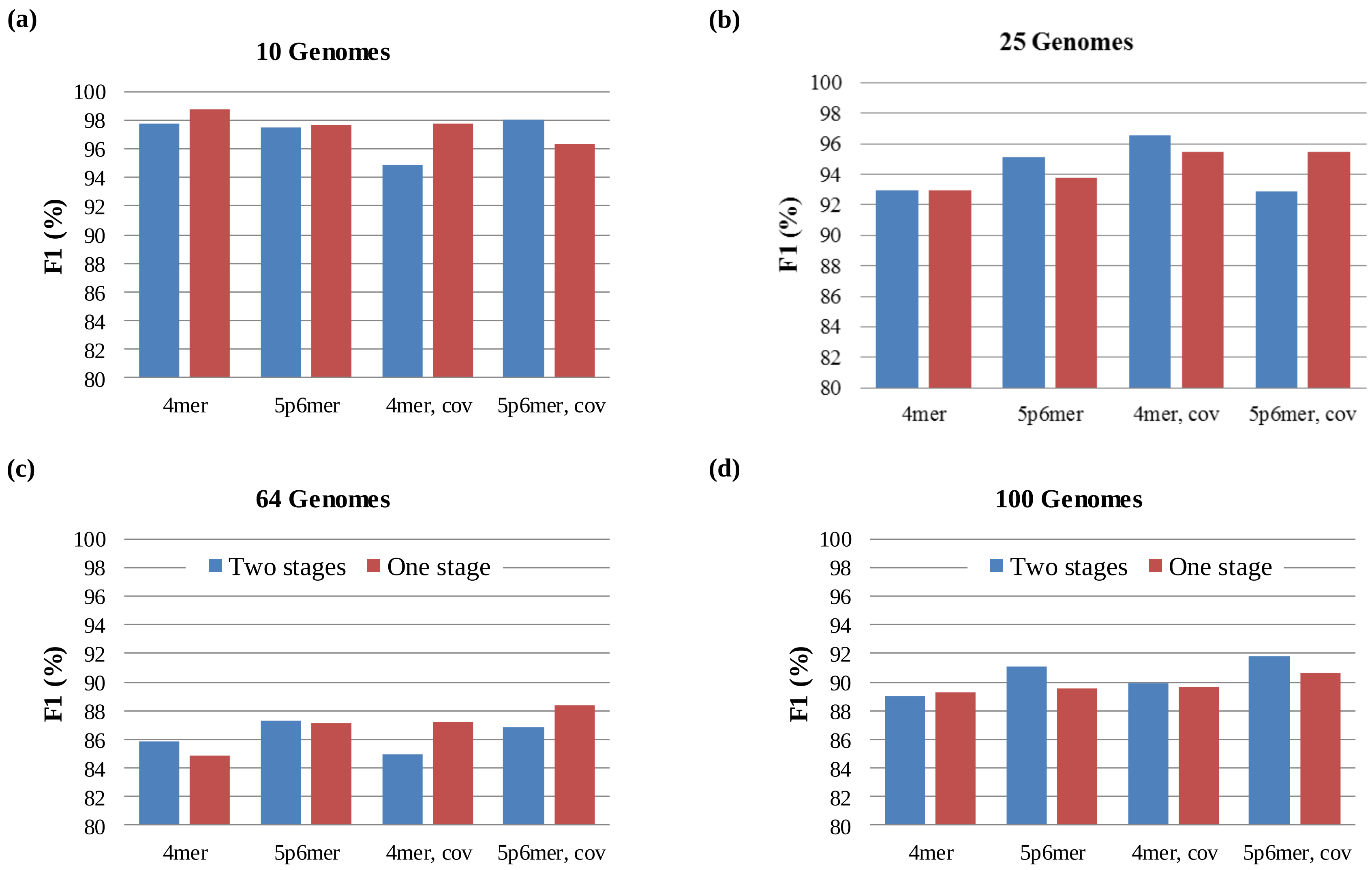

Figure S2 Effects of parameter settings in MyCC.

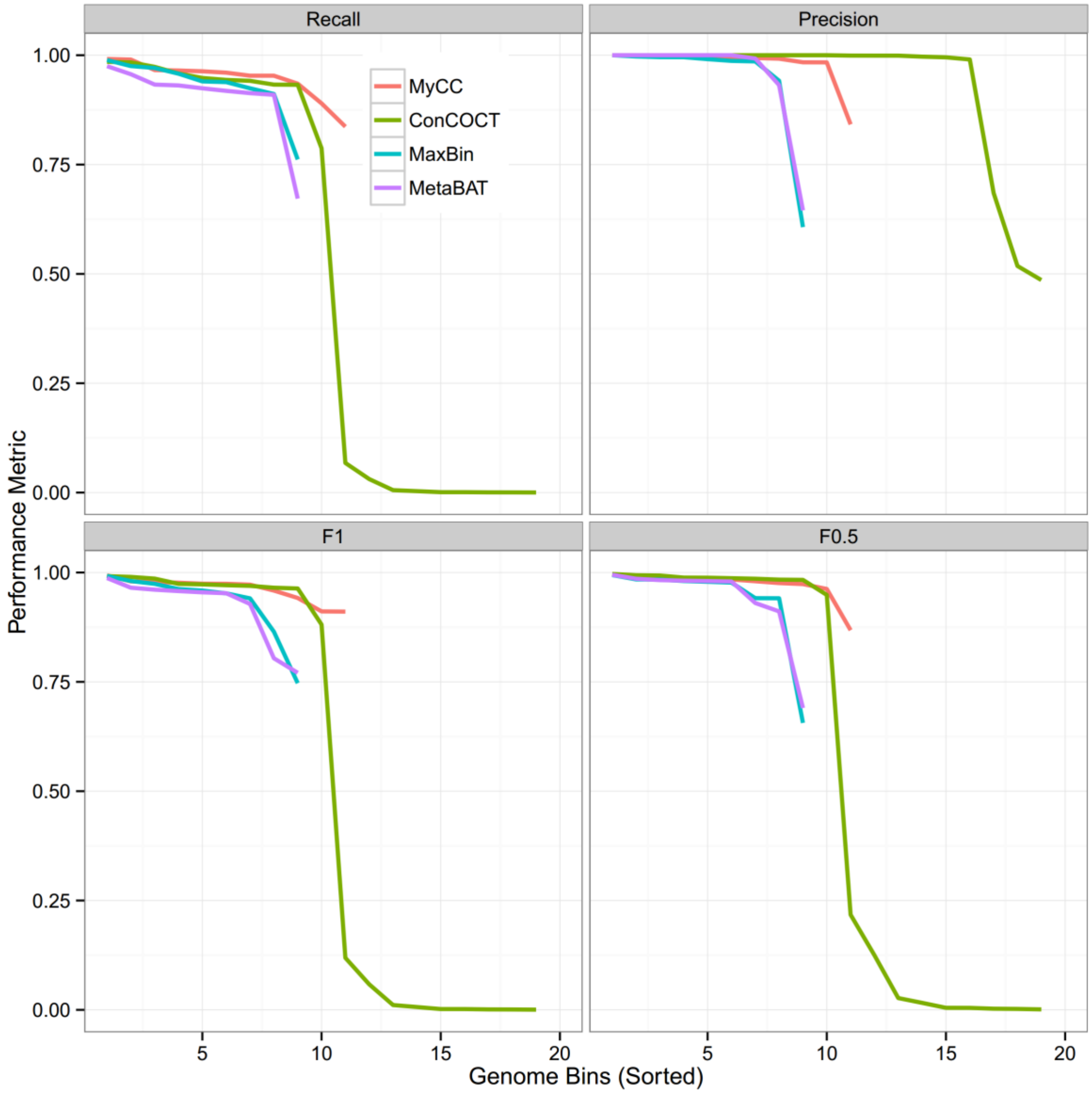

Figure S3 Relative performance of various binning tools evaluated by benchmark.R on the dataset of 10 genomes. Binning results are available at http://sourceforge.net/projects/sb2nhri/files/MyCC/Data/benchmark/10s.zip.

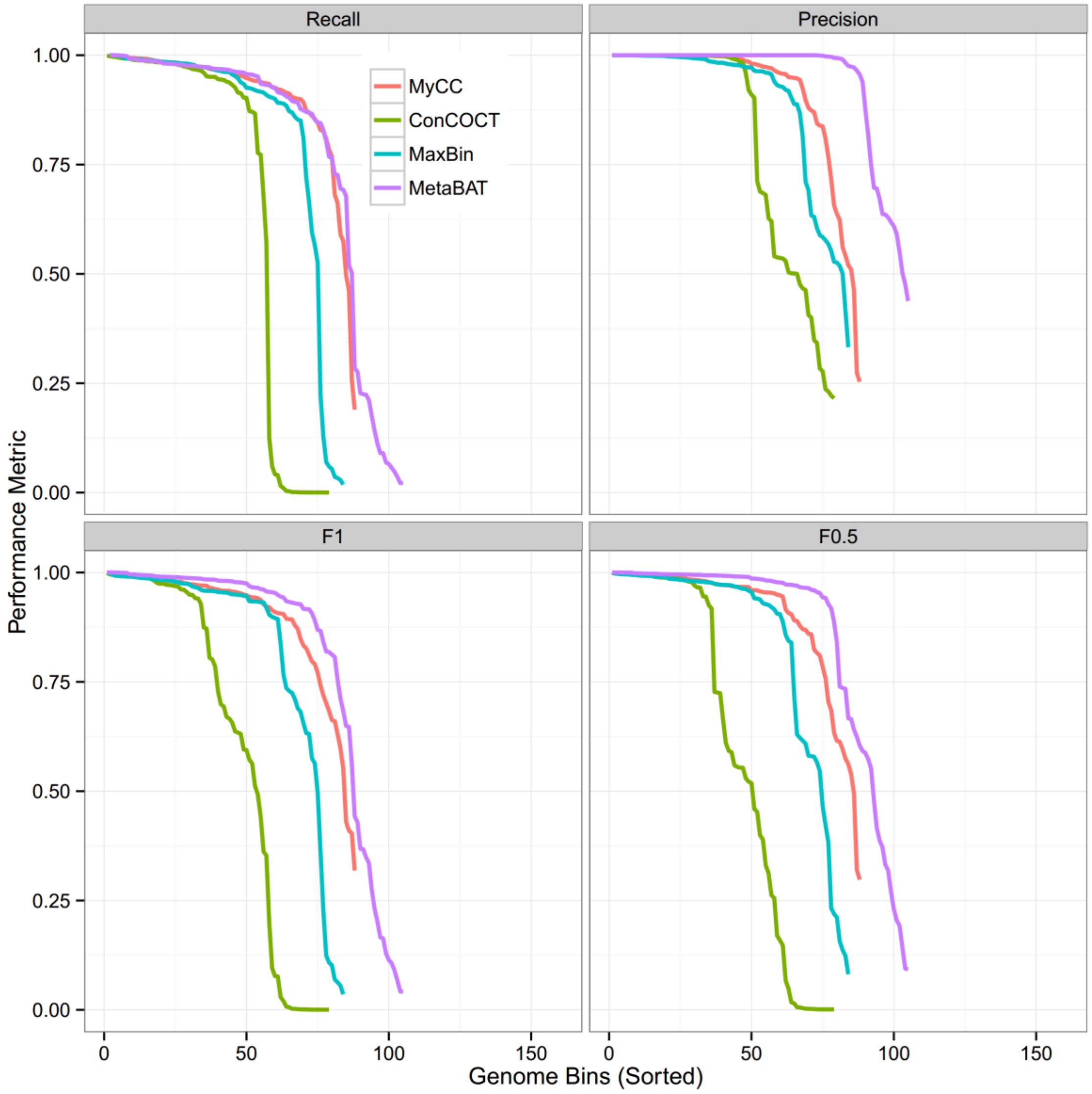

Figure S4 Relative performance of various binning tools evaluated by benchmark.R on the dataset of 100 genomes. Binning results are available at http://sourceforge.net/projects/sb2nhri/files/MyCC/Data/benchmark/100s.zip

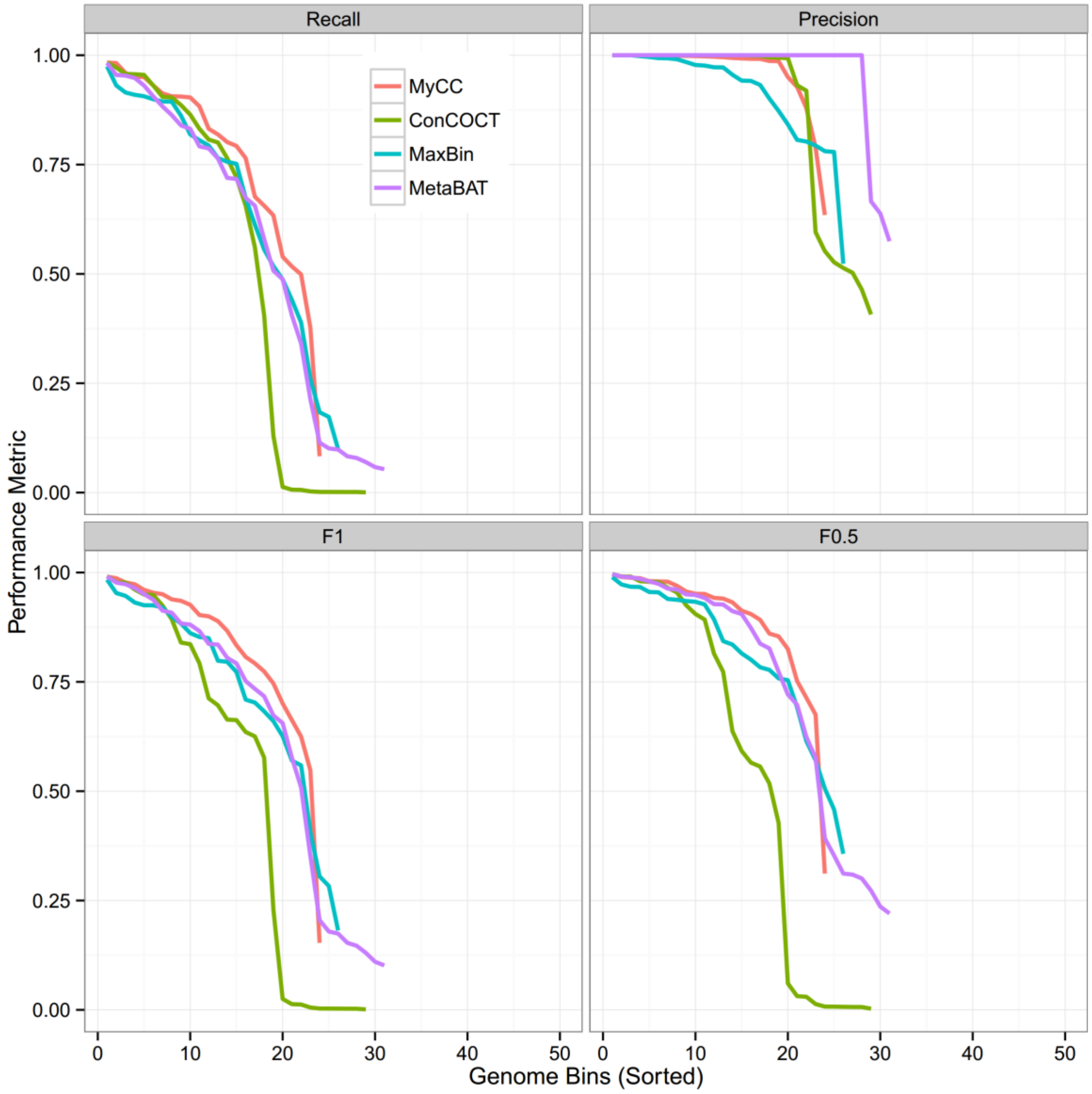

Figure S5 Relative performance of various binning tools evaluated by benchmark.R on the dataset of 25 genomes. Binning results are available at http://sourceforge.net/projects/sb2nhri/files/MyCC/Data/benchmark/25s.zip.

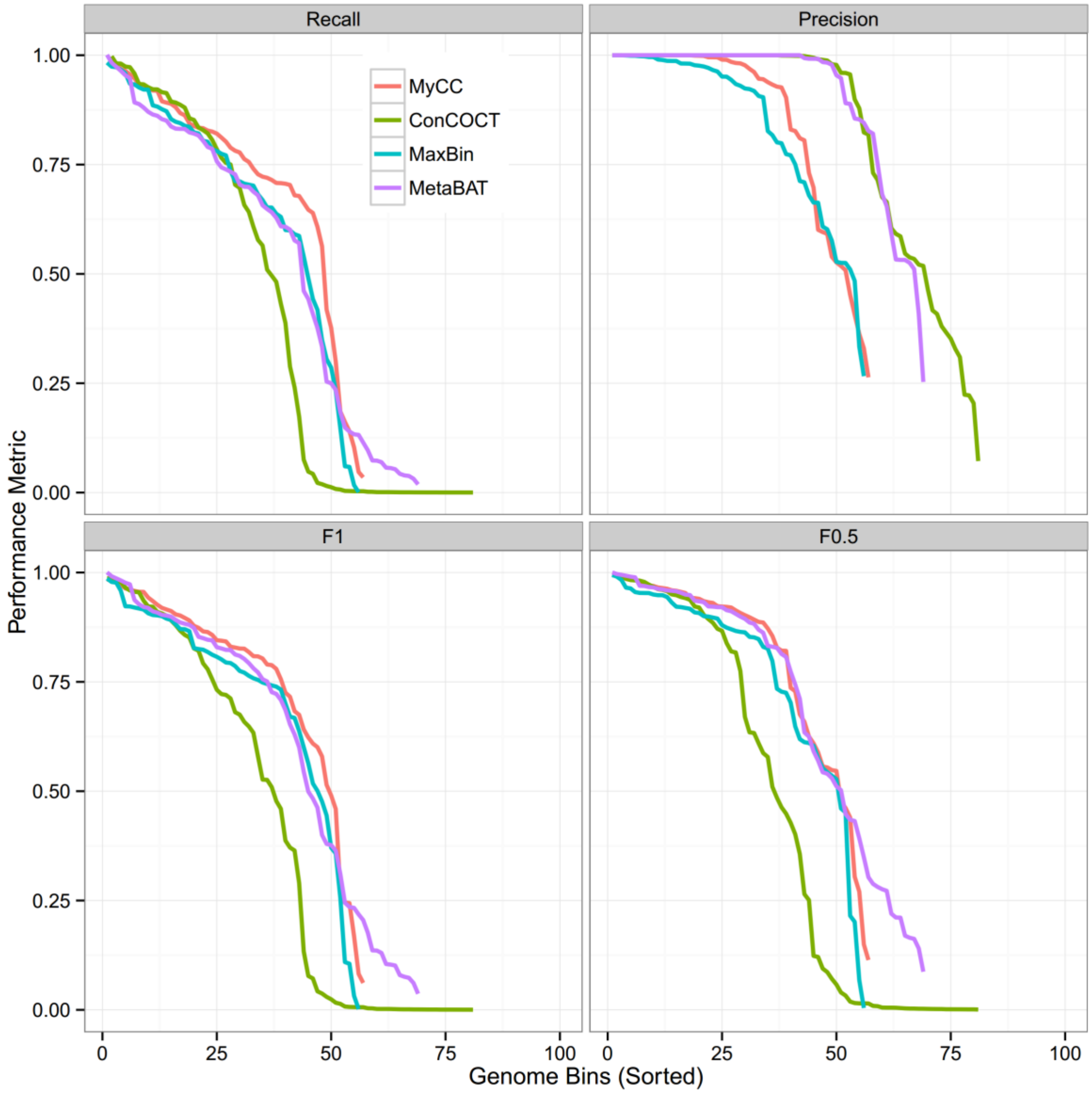

Figure S6 Relative performance of various binning tools evaluated by benchmark.R on the dataset of 64 genomes. Binning results are available at http://sourceforge.net/projects/sb2nhri/files/MyCC/Data/benchmark/64s.zip.

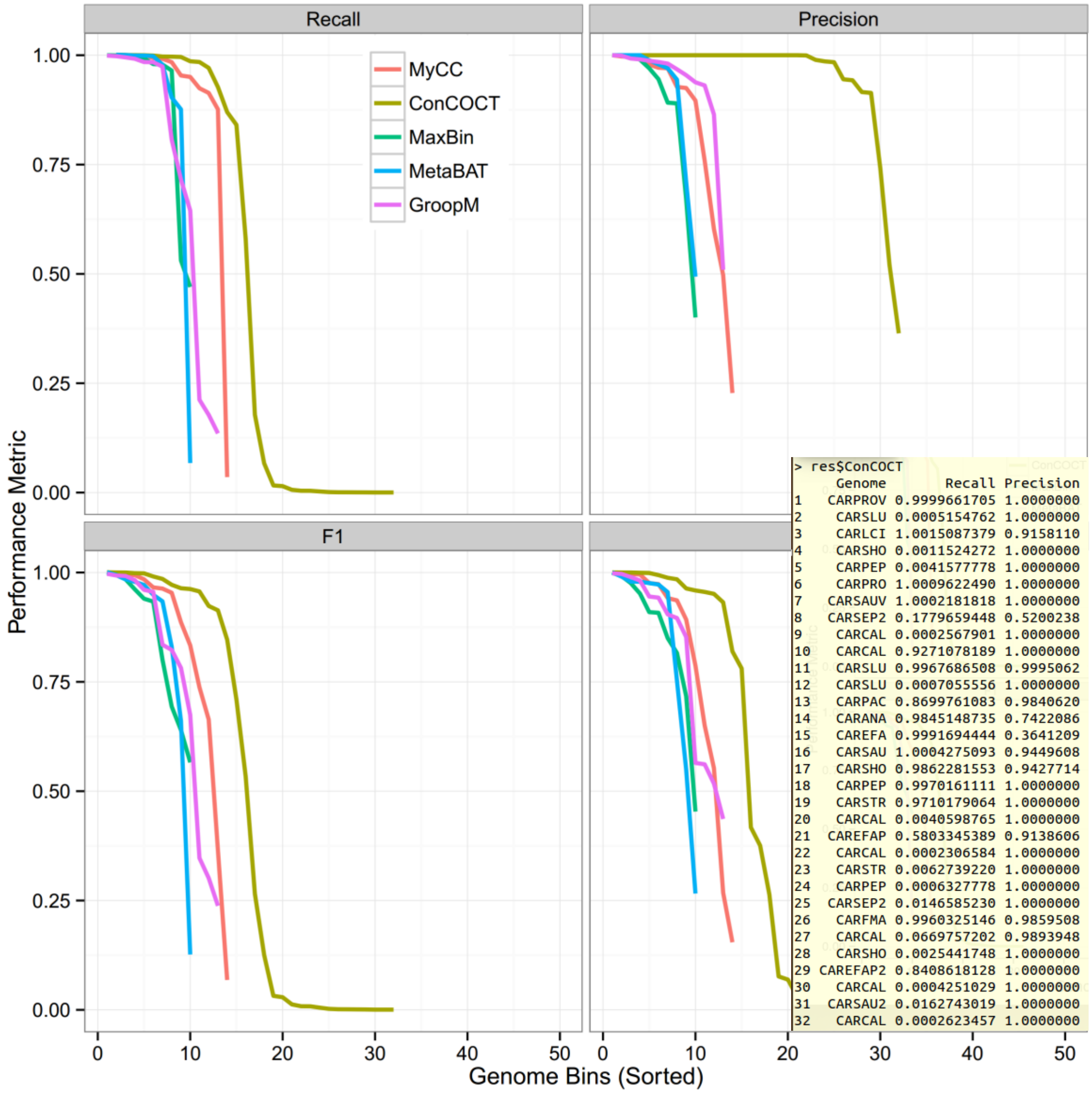

Figure S7 Relative performance of various binning tools evaluated by benchmark.R on the Sharon's dataset. Binning results are available at http://sourceforge.net/projects/sb2nhri/files/MyCC/Data/benchmark/Sharon.zip. Please note that CONCOCT produced many small bins containing only several contigs, which result in high-precision but low-recall binning performance.

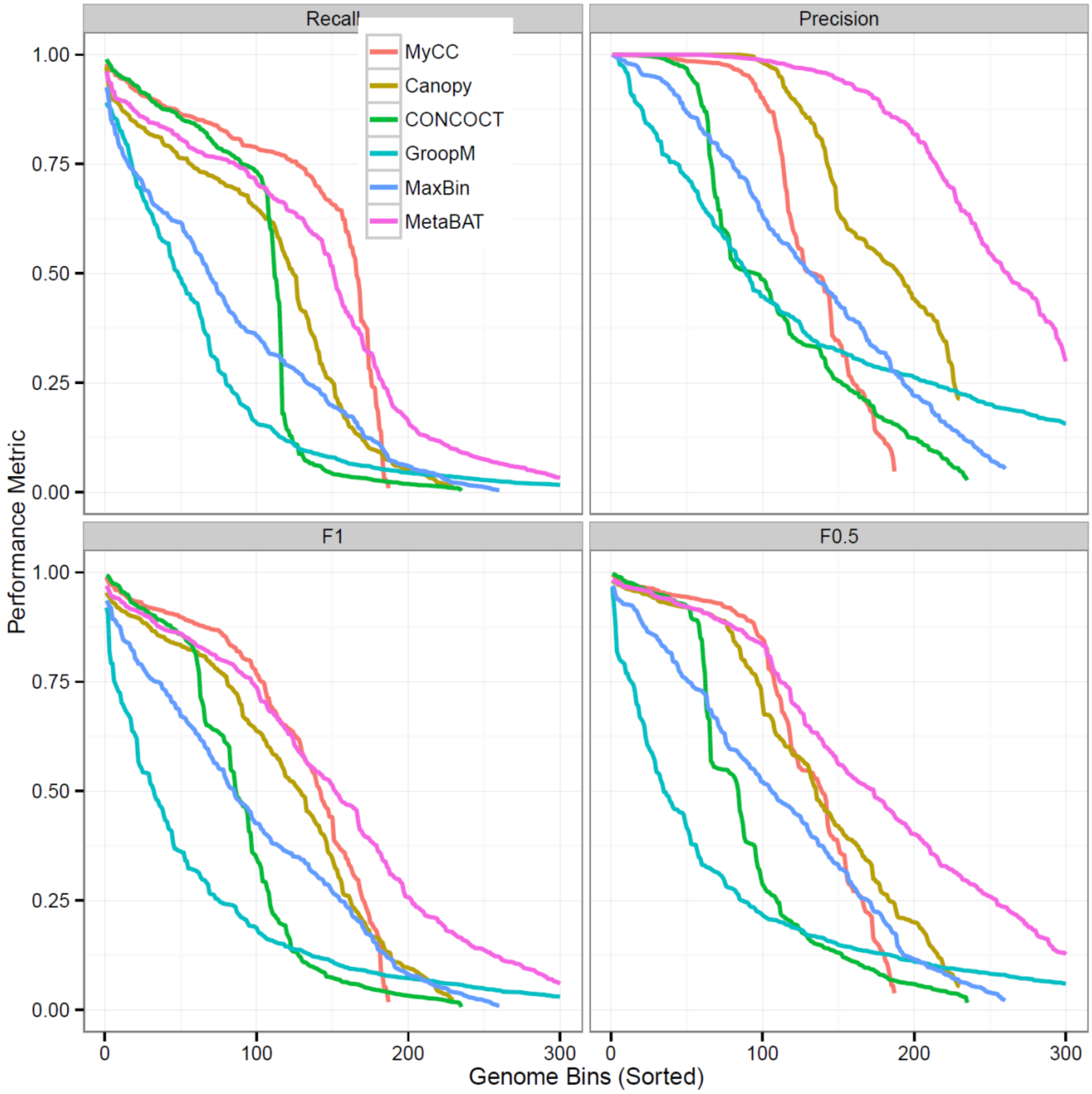

Figure S8 Relative performance of various binning tools evaluated by benchmark.R on the MetaHIT dataset.

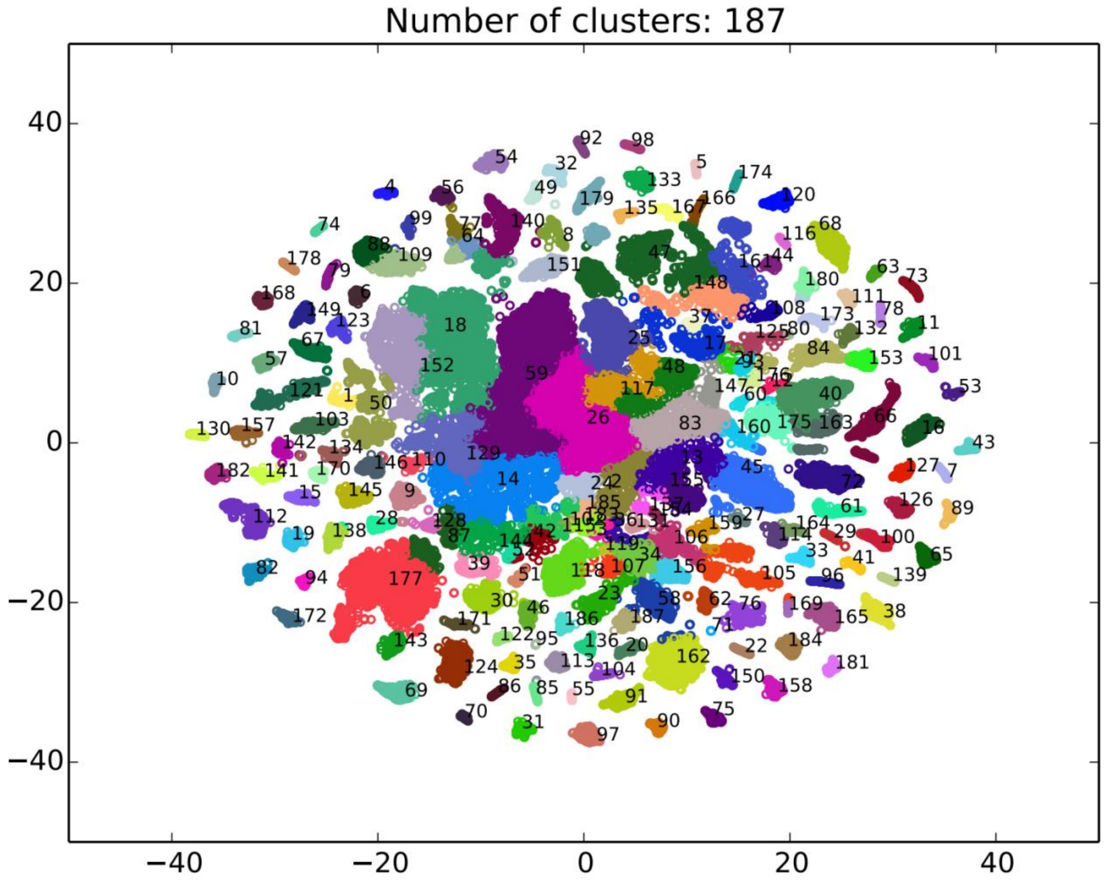

Figure S9 Visualization of metagenomes derived from the 264 MetaHIT human gut metagenome data.

Table S1 Estimation of completeness, contamination and strain heterogeneity provided by CheckM

CONCOCT				
Bin ID	Markder lineage	Completeness	Contamination	Heterogeneity
Cluster_22	root (UID1)	100	204.17	33.92
Cluster_23	g__Staphylococcus (UID301)	99.51	2.91	60
**Cluster_25**	**o__Clostridiales (UID1120)**	**98.95**	**0**	**0**
**Cluster_14**	**o__Actinomycetales (UID1530)**	**97.86**	**0**	**0**
**Cluster_24**	**g__Staphylococcus (UID298)**	**95.39**	**0.57**	**0**
Cluster_19	g__Staphylococcus (UID298)	84.07	0.02	0
Cluster_11	f__Leuconostocaceae (UID486)	45.08	0.23	0
Cluster_18	k__Archaea (UID2)	31.63	8.54	0
Cluster_3	o__Clostridiales (UID1125)	28.73	0	0
Cluster_26	g__Streptococcus (UID576)	16.31	0.33	0
Cluster_20	k__Bacteria (UID203)	5.49	0	0
Cluster_21	k__Bacteria (UID203)	4.73	0	0
Cluster_4	k__Archaea (UID2)	3.74	0	0
19 Clusters	root (UID1)	0	0	0
GroopM				
Bin ID	Markder lineage	Completeness	Contamination	Heterogeneity
myDB_bin_10	root (UID1)	100	104.17	96.55
**myDB_bin_11**	**g__Staphylococcus (UID301)**	**99.51**	**0.08**	**0**
**myDB_bin_16**	**o__Lactobacillales (UID544)**	**99.25**	**0**	**0**
**myDB_bin_9**	**o__Actinomycetales (UID1530)**	**97.86**	**0**	**0**
myDB_bin_6	g__Staphylococcus (UID298)	95.81	1.75	16.67
myDB_bin_4	g__Staphylococcus (UID298)	82.52	3.03	0
myDB_bin_2	k__Archaea (UID2)	34.43	9.48	5.88
myDB_bin_7	f__Leuconostocaceae (UID486)	24.66	0	0
myDB_bin_1	o__Clostridiales (UID1125)	24.37	0.15	0
myDB_bin_13	g__Streptococcus (UID576)	13.81	0.53	0
myDB_bin_17	k__Bacteria (UID203)	13.79	0	0
myDB_bin_19	k__Bacteria (UID203)	3.45	0	0
myDB_bin_18	root (UID1)	0	0	0
MaxBin2				
Bin ID	Markder lineage	Completeness	Contamination	Heterogeneity
maxbin.008	k__Bacteria (UID203)	100	24.37	0
**maxbin.005**	**g__Staphylococcus (UID301)**	**99.51**	**0.08**	**0**

**maxbin.001**	**o__Lactobacillales (UID544)**	**99.25**	**0**	**0**
**maxbin.002**	**o__Clostridiales (UID1120)**	**98.95**	**0**	**0**
maxbin.007	g__Staphylococcus (UID294)	97.91	3.37	81.82
maxbin.006	g__Staphylococcus (UID294)	97	1.12	100
maxbin.009	g__Staphylococcus (UID298)	84.9	2.53	0
maxbin.010	k__Bacteria (UID203)	72.81	37.95	0
maxbin.004	o__Actinomycetales (UID1530)	66.94	0	0
maxbin.003	k__Bacteria (UID203)	22.41	0	0

MetaBAT

Bin ID	Markder lineage	Completeness	Contamination	Heterogeneity
myOutput.4	k__Bacteria (UID203)	100	37.96	0
myOutput.2	root (UID1)	100	108.33	93.44
myOutput.5	g__Staphylococcus (UID301)	99.51	2.91	60
**myOutput.1**	**o__Lactobacillales (UID544)**	**99.25**	**0**	**0**
**myOutput.3**	**o__Clostridiales (UID1120)**	**98.95**	**0**	**0**
**myOutput.6**	**g__Staphylococcus (UID298)**	**95.39**	**0.57**	**0**
myOutput.9	g__Staphylococcus (UID298)	84.1	0.02	0
myOutput.10	f__Leuconostocaceae (UID486)	37.7	0	0
myOutput.7	k__Archaea (UID2)	31.55	8.54	0
myOutput.8	root (UID1)	0	0	0

MyCC

Bin ID	Markder lineage	Completeness	Contamination	Heterogeneity
Cluster.8	root (UID1)	100	104.17	96.55
**Cluster.10**	**g__Staphylococcus (UID301)**	**99.51**	**0.08**	**0**
**Cluster.12**	**o__Lactobacillales (UID544)**	**99.25**	**0**	**0**
**Cluster.1**	**o__Clostridiales (UID1120)**	**98.95**	**0**	**0**
**Cluster.7**	**o__Actinomycetales (UID1530)**	**97.86**	**0**	**0**
**Cluster.13**	**g__Staphylococcus (UID298)**	**95.39**	**0.57**	**0**
Cluster.6	g__Staphylococcus (UID298)	78.73	0	0
Cluster.2	f__Leuconostocaceae (UID486)	45.08	0.23	0
Cluster.14	o__Clostridiales (UID1125)	38.34	3.48	0
Cluster.4	k__Archaea (UID2)	34.43	9.48	5.88
Cluster.11	k__Bacteria (UID203)	7.21	0	0
Cluster.9	g__Staphylococcus (UID298)	5.36	0.02	0
Cluster.3	root (UID1)	4.17	0	0
Cluster.5	k__Bacteria (UID203)	2.66	0	0

Supplementary Note

Install MyCC (Virtual Box for Windows or Mac)

Download Virtual Box

https://www.virtualbox.org/wiki/Downloads

Download the image file of MyCC (MyCC.ova) via

http://sourceforge.net/projects/sb2nhri/files/MyCC/

Open VirtualBox

File -> Import Appliance...

Select the file (MyCC.ova) to import

Or, directly double click on MyCC.ova

Please check the box of "Reinitialize the MAC address of all network cards"

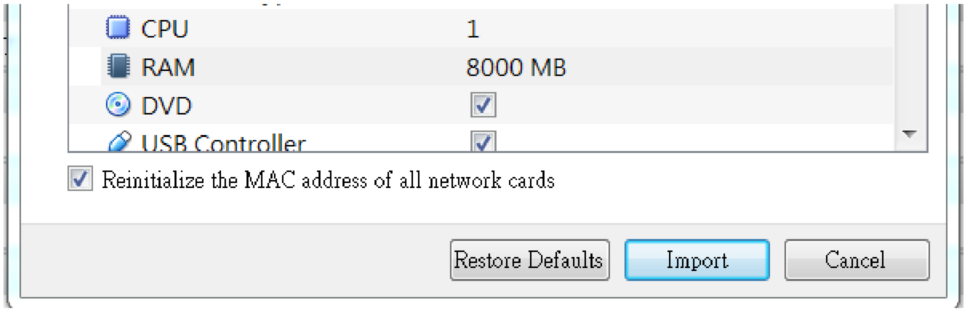

Import

Click on Shared folders to add share (e.g. Data_MyCC in your local computer)

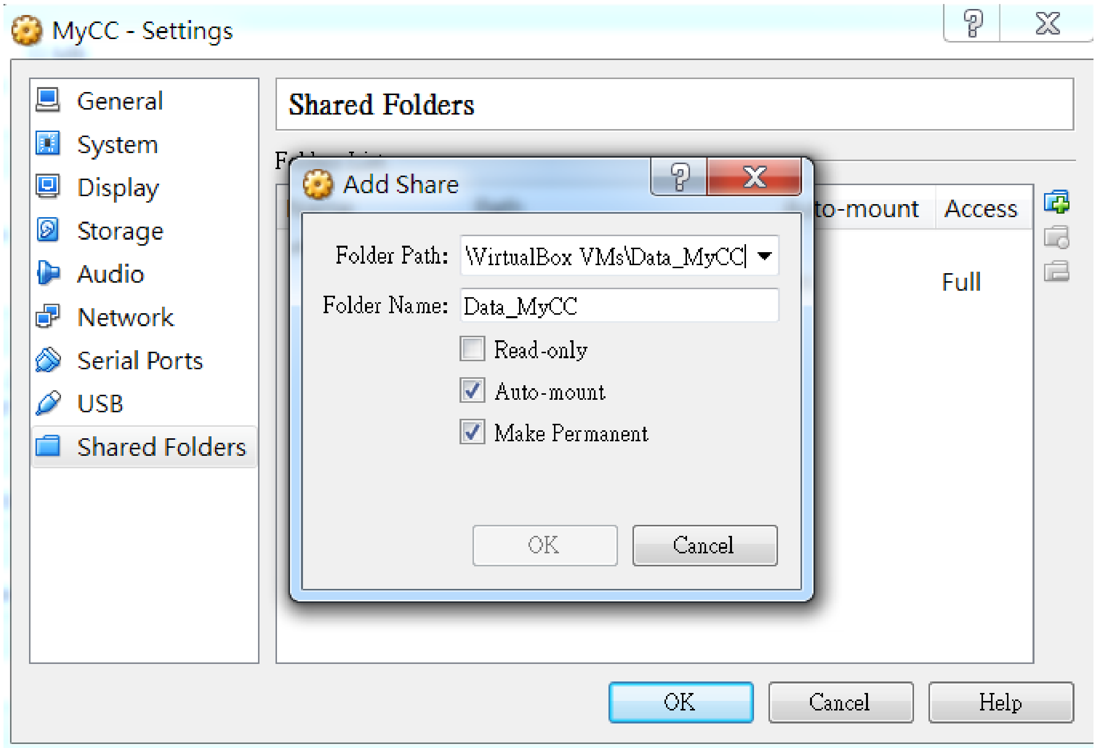

Check Auto-mount and Make-Permanent

OK -> OK

Start

Devices -> Shared Clipboard --> Bidirectional

Open Terminal

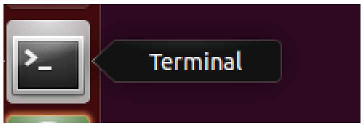

Mount to the shared folder

sudo mount -t vboxsf Data_MyCC MyData

(Password for manager: manager)

Install MyCC (Virtual Box for Linux)

In Ubuntu 15.10:

sudo apt-get install virtualbox

wget http://sourceforge.net/projects/sb2nhri/files/MyCC/MyCC.ova

vboxmanage import MyCC.ova

vboxmanage startvm MyCC

Power off MyCC:

vboxmanage controlvm MyCC poweroff

See https://www.virtualbox.org/manual/ for further information.

Run MyCC

MyCC.py -h

Usage:

MyCC.py [inputfile] [4mer/5mer/56mer default:4mer] [Options]

Options:

-a A file having coverage information.

-t Minimum contig length. [defaults: 1000 bp]

-lt A fraction of contigs for the first-stage clustering. [default: 0.7]

-ct Minimum contig lengh for first stage clustering. [bp]

-meta Change to meta mode of Prodigal. [default: single]

-p Perplexity for BH-SNE. [default: 20, range between 5 and 50]

-pm To set preferences all equal to the median of the other similarities for affinity propagation.

-st Maximum distance for sparse format of affinity propagation. [default: 500]

-mask To mask repetitive sequences.

-keep To keep temporary files.

The 10-Genome, 25-Genome, 64-Genomes, 100-Genome and Sharon's datasets were able to complete within one hour using Intel Xeon CPU E31245 3.30GHz with 4GB RAM. Please note that the following results were produced on the Ubuntu Virtual Machine System, they are slightly different from the results present in Table 1, which were produced on a Centos server with Intel Xeon E7-4820 processors 8-core 2.00 GHz and 256 GB of RAM.

mkdir Run
cd Run

Produce coverage profiles:

Please note that we used Bowtie 2 to produce BAM files. Then we run MetaBAT to produce a depth file: jgi_summarize_bam_contig_depths --outputDepth depth.txt *.bam. We took the columns of contigName and *.bam in the file of depth.txt to produce our depth file.

Dataset: 10 Genomes

A RayMeta assembly: raymeta_10.fasta

A filtered assembly: 10s.fasta (contigs>=1000 bp, Header without space)

Binning gold standard: 10s.spe.txt

A coverage file: 10s.depth.txt

wget http://sourceforge.net/projects/sb2nhri/files/MyCC/Data/10s.zip

unzip 10s.zip

cd 10s

MyCC.py 10s.fasta

```
manager@sb:~/Run/10s$ MyCC.py 10s.fasta
20151204_1437
4mer
1_rename.py /home/manager/Run/10s/10s.fasta 1000
Seqs >= 1000 : 2185
Minimum contig lengh for first stage clustering: 3786
run Prodigal.
/opt/prodigal.linux -i My.fa -a gene.aa -d gene.nuc -f gbk -o output -s potentia
l_genes.txt
run fetchMG.
run UCLUST.
Get Feature.
2_GetFeatures_4mer.py for fisrt stage clustering
2_GetFeatures_4mer.py for second stage clustering
3_GetMatrix.py 3786 for fisrt stage clustering
 1538 contigs entering first stage clustering
Clustering...
1_bhsne.py 20
2_ap.py /opt/ap 500 0
Cluster Correction.
to Split and Merge.
1_ClusterCorrection_Split.py 40 2
2_ClusterCorrection_Merge.py 40
Get contig by cluster.
20151204_1443
```

MyCC outputs a folder named yyyymmdd_hhmm_kmer_lt (*e.g.*, 20151204_1437_4mer_0.7). The folder contains binning sequences for each cluster in FASTA format (**Cluster.N.fasta**), a summary file (**Cluster.summary**), a visualization plot (**MyCluster.pdf**) and a coordinate file (**MyCluster.coords**).

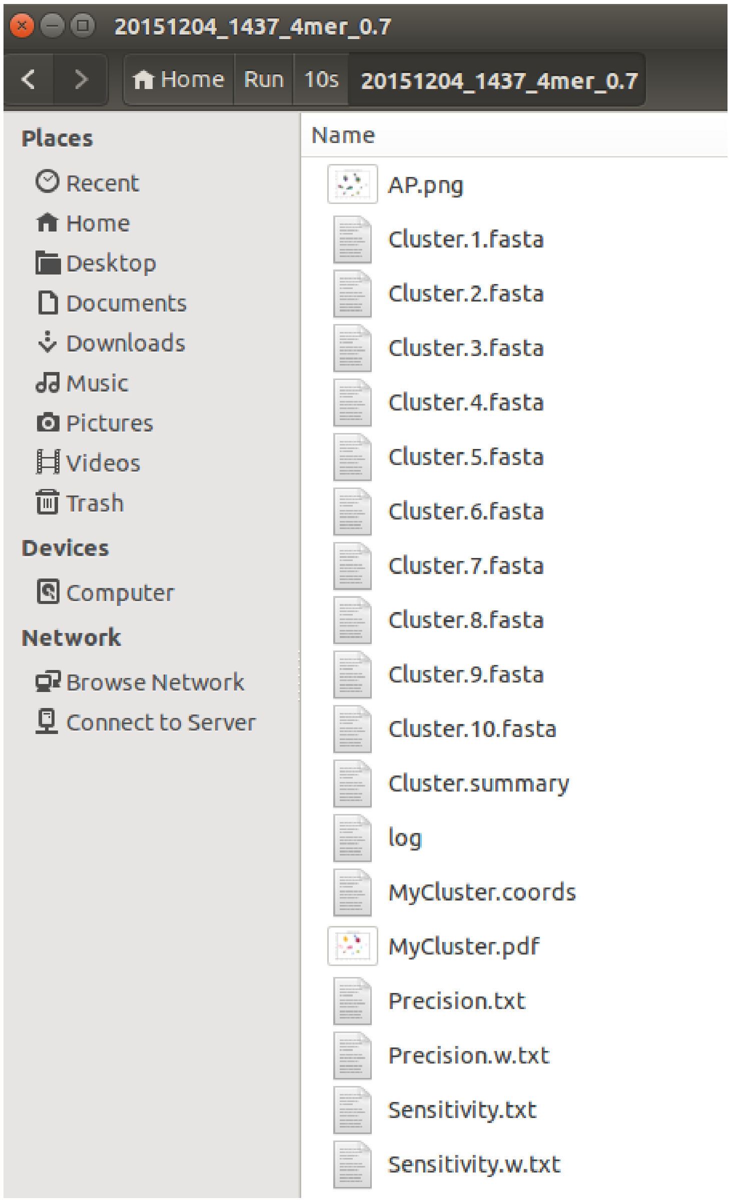

Cluster.summary ×

```
Cluster WholeGenome     N50     NoOfCtg LongestCtgLen   AvgLenOfCtg     Cogs
Cluster.1.fasta 1901145 7154    359     22997   2657    38
Cluster.2.fasta 4516525 24042   327     103378  6907    37
Cluster.3.fasta 2736452 34074   164     91793   8424    37
Cluster.4.fasta 4220229 83088   109     294361  19374   41
Cluster.5.fasta 3540046 43992   155     145092  11579   12
Cluster.6.fasta 1696113 56131   70      203033  12900   37
Cluster.7.fasta 1707337 74948   52      202223  17699   38
Cluster.8.fasta 5316241 19489   465     114410  5716    39
Cluster.9.fasta 4753388 20166   405     120224  5885    11
Cluster.10.fasta        2760436 61646   79      206967  18251   37
```

Description of Cluster.summary

Cluster: cluster name

WholeGenome: the total length of contigs in a cluster

N50

NoOfCtg: the number of contigs in a cluster

LongestCtgLen: the length of the longest contig

AvgLenOfCtg: the average length of contigs in a cluster

Cogs: the number of marker genes in a cluster

MyCluster.pdf:

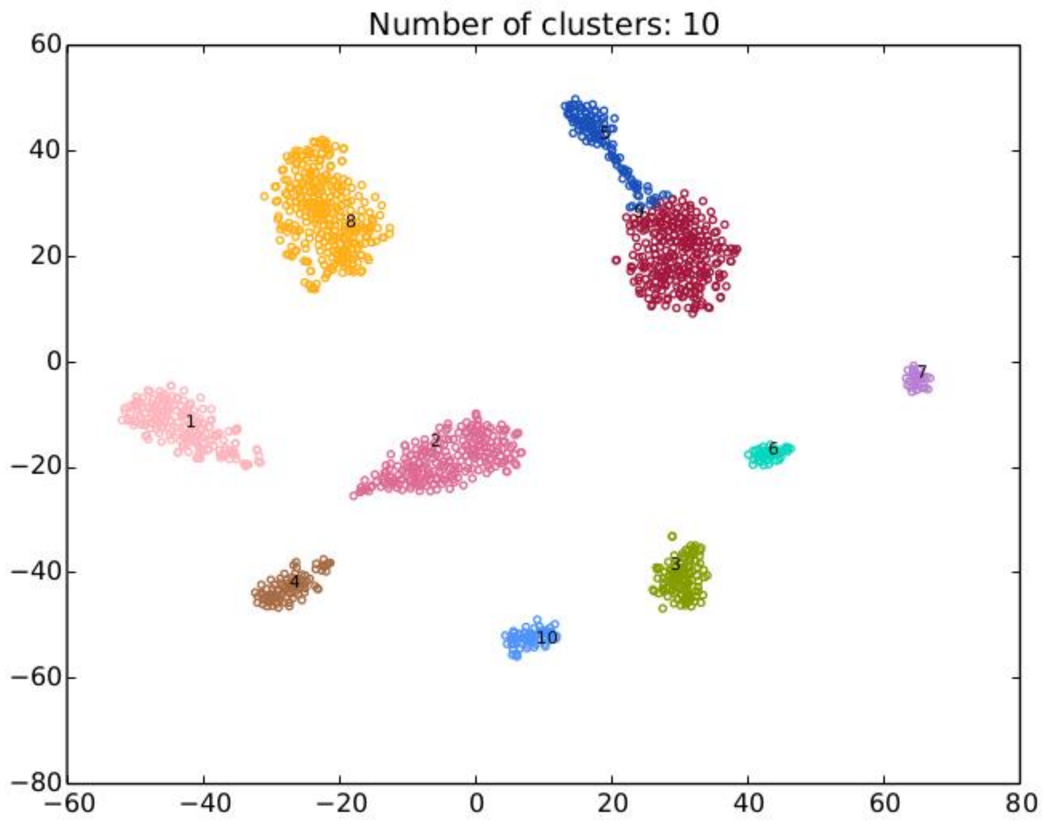

Evaluate:

Evaluate.py -h

Please input two files containing reference assignment and assembly, followed by target name (e.g., fasta).

Usage:

Evaluate.py [reference assignment] [assembly] [target name] [options]

option:

-split To split header by space.

-plot To plot references based on the MyCluster.coords.
-h Help.

cd 20151204_1437_4mer_0.7/
Evaluate.py ../10s.spe.txt ../10s.fasta fasta
(Please note that "-split" is required if the sequence headers contain space. For example, Evaluate.py ../10s.spe.txt ../raymeta_10.fasta fasta -split)

```
manager@sb:~/Run/10s$ cd 20151204_1437_4mer_0.7/
manager@sb:~/Run/10s/20151204_1437_4mer_0.7$ Evaluate.py ../10s.spe.txt ../10s.fas
ta fasta
No. of reference genomes: 10
No. of bins in evaluation: 10
No. of sequences assigned reference: 2172
No. of binned sequences: 2185
Precision: 0.944291, 0.974709
Sensitivity: 0.944291, 0.974709
```

Please note that the first value for precision/sensitivity only takes the number of contigs into account. However, we emphasize on the precision and sensitivity based on the total length of contigs in a cluster (the second value), as described in the manuscript.
Assume there are N genomes in the dataset, which were binned into M clusters. The overall precision and recall (sensitivity) are calculated as

$$\text{Precision}\ (\%) = \frac{\sum_{i=1}^{M} \max_{j} S_{ij}}{\sum_{i=1}^{M} \sum_{j=1}^{N} S_{ij}} \times 100$$

$$\text{Recall}\ (\%) = \frac{\sum_{j=1}^{N} \max_{i} S_{ij}}{\sum_{i=1}^{M} \sum_{j=1}^{N} S_{ij} + \sum \mathit{length\ of\ unbinned\ sequences}} \times 100$$

in which S_{ij} indicates the total length of contigs in a cluster *i* corresponding to a reference genome *j*.
Precision based on the number of contigs: (listed in **Precision.txt**)

```
Precision.txt
Cluster.10.fasta:0.860759(68/79)        NC_003112:4     NC_009637:3     NC_008555:68    NC_009641:4
Cluster.1.fasta:1.000000(359/359)       NC_003112:359
Cluster.3.fasta:0.902439(148/164)       NC_009637:1     NC_003112:2     NC_009641:148   NC_008555:12    NC_007779:1
Cluster.6.fasta:0.928571(65/70) NC_003112:3     NC_009637:65    NC_007779:2
Cluster.9.fasta:1.000000(405/405)       NC_005296:405
Cluster.7.fasta:0.923077(48/52) NC_006582:2     NC_003112:1     NC_008555:1     NC_008011:48
Cluster.8.fasta:0.955947(434/454)       NC_006582:7     NC_003112:7     NC_009637:1     NC_008011:1     NC_008555:3     NC_010546:434   NC_007779:1
Cluster.2.fasta:0.969419(317/327)       NC_003112:10    NC_007779:317
Cluster.4.fasta:0.898148(97/108)        NC_006582:97    NC_010546:1     NC_003112:6     NC_008555:1     NC_007779:3
Cluster.5.fasta:0.714286(110/154)       NC_007404:110   NC_005296:42    NC_009637:2
```

Overall precision:
(68+359+148+65+405+48+434+317+97+110)/(79+359+164+70+405+52+454+327+108+154)=2051/2172=0.944291

Precision based on the total length of contigs: (listed in **Precision.w.txt)**

```
Precision.w.txt
Cluster.10.fasta:0.983843(2715835/2760436)	NC_003112:7851	NC_009637:25013	NC_008555:2715835	NC_009641:11737
Cluster.1.fasta:1.000000(1901145/1901145)	NC_003112:1901145
Cluster.3.fasta:0.983665(2691753/2736452)	NC_009637:1283	NC_003112:3801	NC_009641:2691753	NC_008555:36512	NC_007779:3103
Cluster.6.fasta:0.996210(1689685/1696113)	NC_003112:4113	NC_009637:1689685	NC_007779:2315
Cluster.9.fasta:1.000000(4753388/4753388)	NC_005296:4753388
Cluster.7.fasta:0.996889(1702026/1707337)	NC_006582:2012	NC_003112:2284	NC_008555:1015	NC_008011:1702026
Cluster.8.fasta:0.993817(5167087/5199234)	NC_006582:10460	NC_003112:11682	NC_009637:1638	NC_008011:1774	NC_008555:5591	NC_010546:5167087	NC_007779:1002
Cluster.2.fasta:0.993766(4488370/4516525)	NC_003112:28155	NC_007779:4488370
Cluster.4.fasta:0.993860(4132275/4157805)	NC_006582:4132275	NC_010546:3251	NC_003112:8096	NC_008555:1505	NC_007779:12678
Cluster.5.fasta:0.816875(2884905/3531634)	NC_007404:2884905	NC_005296:643560	NC_009637:3169
```

Overall precision:

(2715835+1901145+2691753+1689685+4753388+1702026+5167087+4488370+4132275+2884905)/(2760436+1901145+2736452+1696113+4753388+1707337+5199234+4516525+4157805+3531634)=32126469/32960069=0.974709

Similarly, we set one stage by specifying “-lt 1”

MyCC.py 10s.fasta -lt 1

```
manager@sb:~/Run/10s$ MyCC.py 10s.fasta -lt 1
20151204_1500
4mer
1_rename.py /home/manager/Run/10s/10s.fasta 1000
Seqs >= 1000 : 2185
Minimum contig lengh for first stage clustering: 1001
run Prodigal.
/opt/prodigal.linux -i My.fa -a gene.aa -d gene.nuc -f gbk -o output -s potential_
genes.txt
run fetchMG.
run UCLUST.
Get Feature.
2_GetFeatures_4mer.py for fisrt stage clustering
2_GetFeatures_4mer.py for second stage clustering
3_GetMatrix.py 1001 for fisrt stage clustering
 2185 contigs entering first stage clustering
Clustering...
1_bhsne.py 20
2_ap.py /opt/ap 500 0
Cluster Correction.
to Split and Merge.
1_ClusterCorrection_Split.py 40 2
2_ClusterCorrection_Merge.py 40
Get contig by cluster.
20151204_1506
```

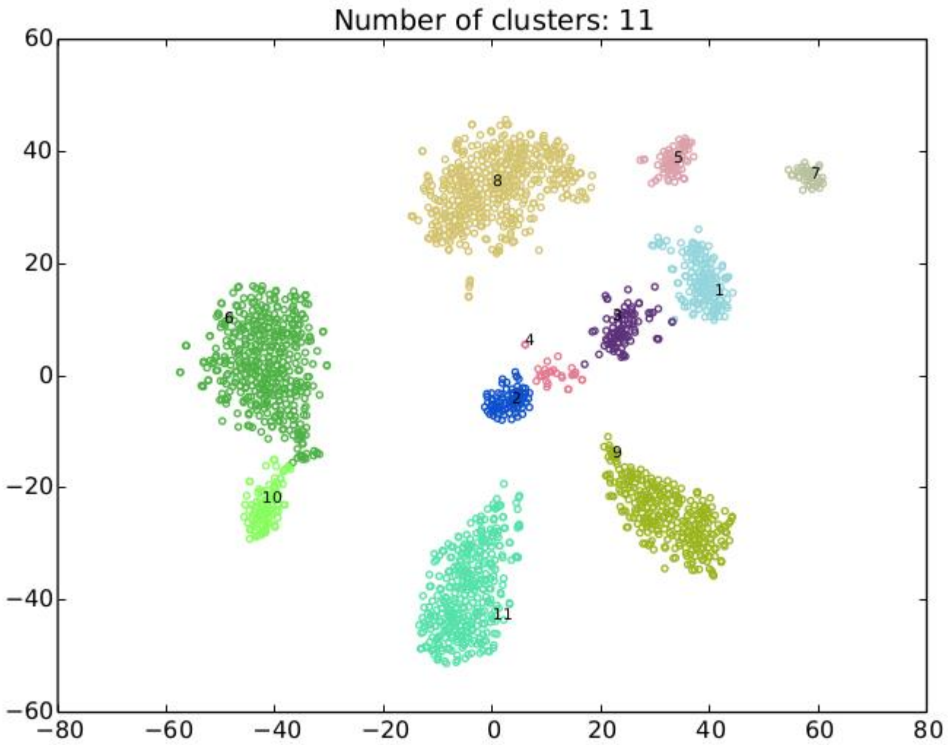

cd 20151204_1500_4mer_1.0/

Evaluate.py ../10s.spe.txt ../10s.fasta fasta -plot

```
manager@sb:~/Run/10s/20151204_1500_4mer_1.0$ Evaluate.py ../10s.spe.txt ../10s.fas
ta fasta -plot
No. of reference genomes: 10
No. of bins in evaluation: 11
No. of sequences assigned reference: 2172
No. of binned sequences: 2185
Precision: 0.954880, 0.987968
Sensitivity: 0.948435, 0.980754
```

By adding the option of "-plot", you can obtain a visualization plot (**MyReference.pdf**) labeled with the names of reference genomes.

MyReference.pdf:

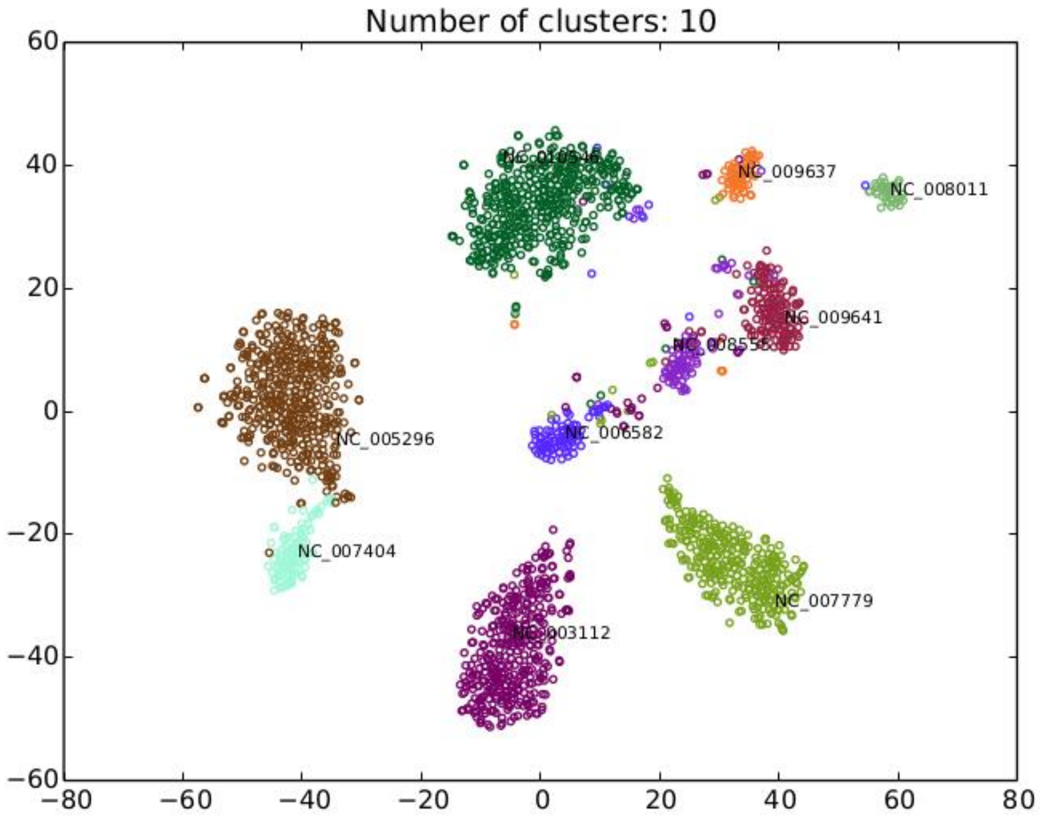

Dataset: 100 Genomes

A RayMeta assembly: Contigs.fasta

A filtered assembly: 100s.fasta (contigs>=1000 bp, Header without space)

A coverage file: 100s.depth.txt

Binning gold standard: 100s.spe.txt

wget http://sourceforge.net/projects/sb2nhri/files/MyCC/Data/100s.zip

unzip 100s.zip

cd 100s

MyCC.py 100s.fasta 56mer -a 100s.depth.txt

```
manager@sb:~/Run/100s$ MyCC.py 100s.fasta 56mer -a 100s.depth.txt
20151207_0925
56mer
1_rename.py /home/manager/Run/100s/100s.fasta 1000
Seqs >= 1000 : 8978
Minimum contig lengh for first stage clustering: 2475
run Prodigal.
/opt/prodigal.linux -i My.fa -a gene.aa -d gene.nuc -f gbk -o output -s potentia
l_genes.txt
run fetchMG.
run UCLUST.
Get Feature.
2_GetFeatures_5mer_p6mer.py for fisrt stage clustering
2_GetFeatures_TimeSeries.py /home/manager/Run/100s/100s.depth.txt
2_GetFeatures_4mer.py for second stage clustering
2_GetFeatures_TimeSeries.py /home/manager/Run/100s/100s.depth.txt
3_GetMatrix.py 2475 for fisrt stage clustering
 6297 contigs entering first stage clustering
Clustering...
1_bhsne.py 20
2_ap.py /opt/ap 500 0
Cluster Correction.
to Split and Merge.
1_ClusterCorrection_Split.py 40 2
2_ClusterCorrection_Merge.py 40
Get contig by cluster.
20151207_1017
```

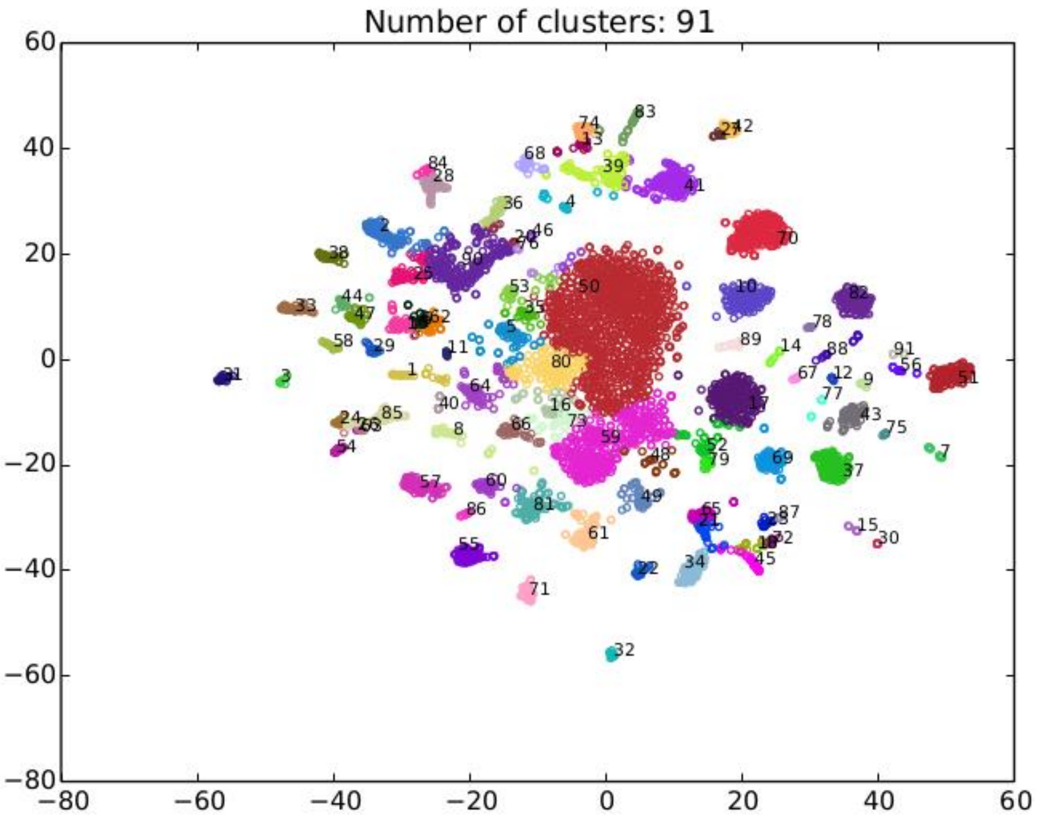

cd 20151207_0925_56mer_0.7_cov/

Evaluate.py ../100s.spe.txt ../100s.fasta fasta -plot

```
manager@sb:~/Run/100s/20151207_0925_56mer_0.7_cov$ Evaluate.py ../100s.spe.txt .
./100s.fasta fasta -plot
No. of reference genomes: 100
No. of bins in evaluation: 91
No. of sequences assigned reference: 8942
No. of binned sequences: 8978
Precision: 0.611832, 0.895118
Sensitivity: 0.782487, 0.940128
[illegible]
```

<u>Dataset: 25 Genomes</u>

Original download site:

http://portal.nersc.gov/dna/RD/Metagenome_RD/MetaBAT/Software/Mockup/

An assembly: assembly.fasta

Binning gold standard: membership.txt

Bam files: library1.bam* and library2.bam*

To generate a depth file from BAM files:

jgi_summarize_bam_contig_depths --outputDepth depth.txt *.bam

We modified the files of depth.txt and membership.txt to My.depth.txt and member.txt, respectively, for MyCC:

A coverage file: My.depth.txt

Binning gold standard: member.txt

wget http://sourceforge.net/projects/sb2nhri/files/MyCC/Data/25s.zip

unzip 25s.zip

cd 25s

MyCC.py assembly.fa -a My.depth.txt

```
manager@sb:~/Run/25s$ MyCC.py assembly.fa -a My.depth.txt
20151207_1023
4mer
1_rename.py /home/manager/Run/25s/assembly.fa 1000
Seqs >= 1000 : 1893
Minimum contig lengh for first stage clustering: 4645
run Prodigal.
/opt/prodigal.linux -i My.fa -a gene.aa -d gene.nuc -f gbk -o output -s potentia
l_genes.txt
run fetchMG.
run UCLUST.
Get Feature.
2_GetFeatures_4mer.py for fisrt stage clustering
2_GetFeatures_TimeSeries.py /home/manager/Run/25s/My.depth.txt
2_GetFeatures_4mer.py for second stage clustering
2_GetFeatures_TimeSeries.py /home/manager/Run/25s/My.depth.txt
3_GetMatrix.py 4645 for fisrt stage clustering
 1328 contigs entering first stage clustering
Clustering...
1_bhsne.py 20
2_ap.py /opt/ap 500 0
Cluster Correction.
to Split and Merge.
1_ClusterCorrection_Split.py 40 2
2_ClusterCorrection_Merge.py 40
Get contig by cluster.
20151207_1034
```

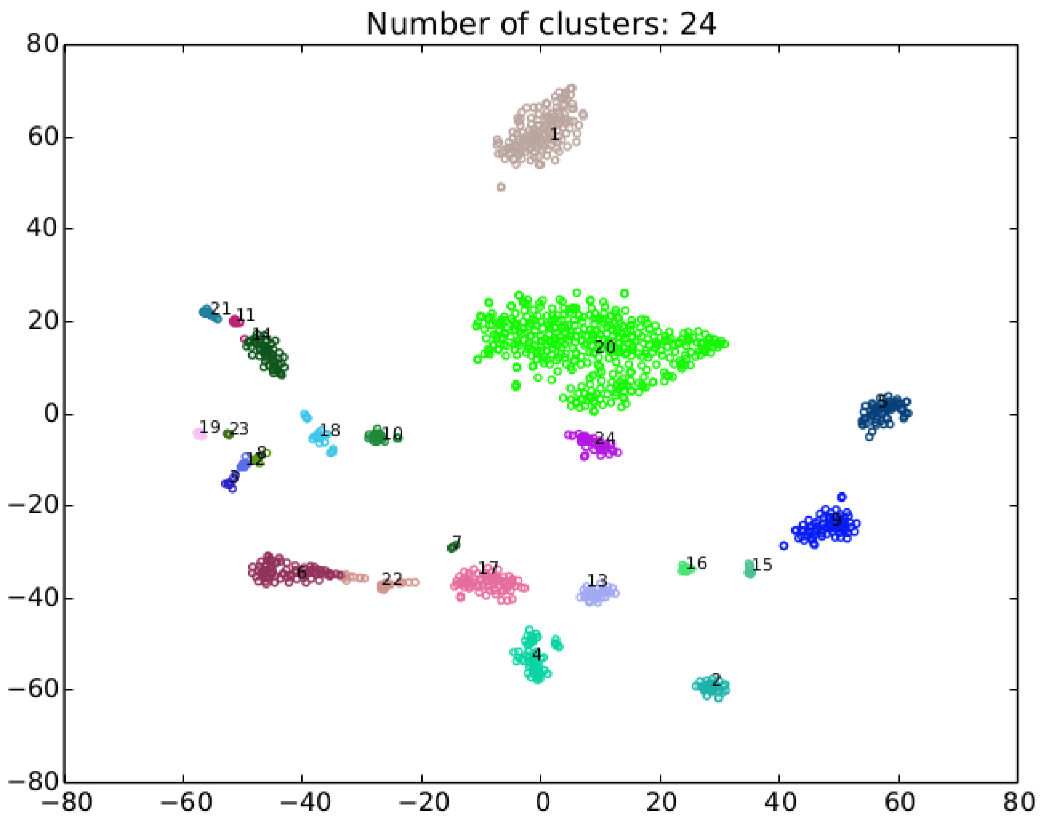

cd 20151207_1023_4mer_0.7_cov

Evaluate.py ../member.txt ../assembly.fa fasta -plot

```
manager@sb:~/Run/25s/20151207_1023_4mer_0.7_cov$ Evaluate.py ../member.txt ../assembly.fa fasta -plot
No. of reference genomes: 25
No. of bins in evaluation: 24
No. of sequences assigned reference: 1893
No. of binned sequences: 1893
Precision: 0.797147, 0.958671
Sensitivity: 0.853143, 0.972830
```

MyReference.pdf:

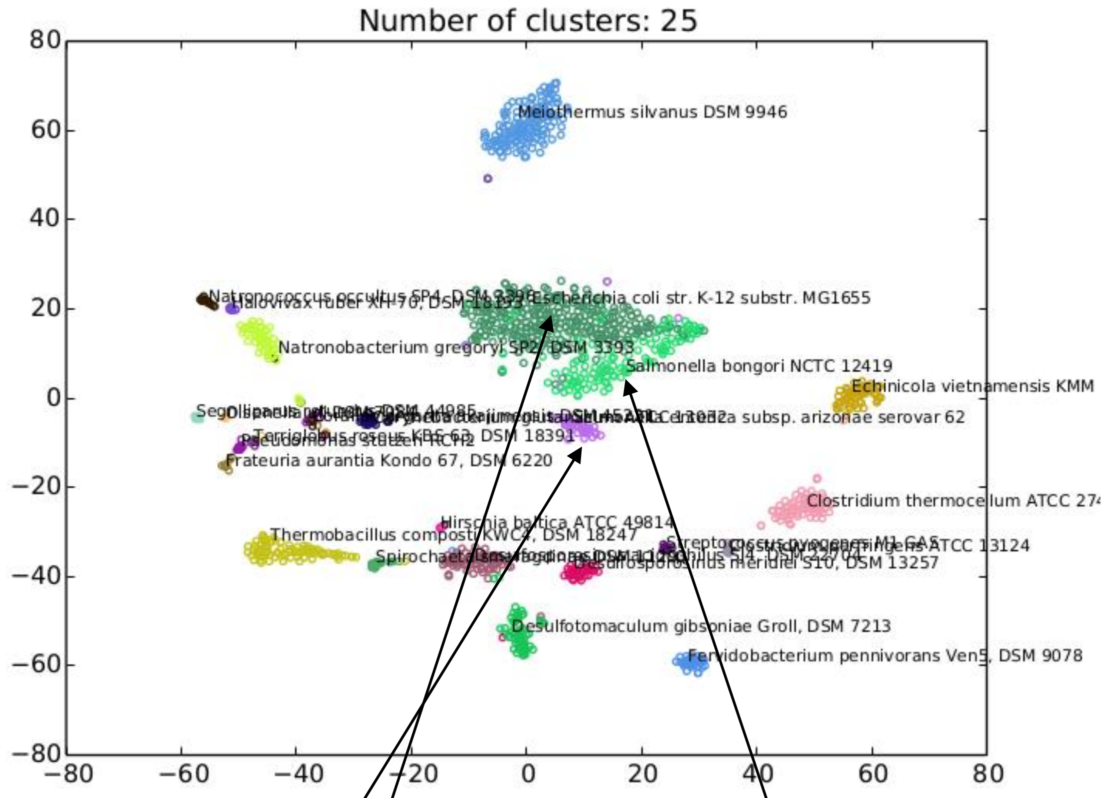

In comparing MyCluster.pdf with MyReference.pdf, we found MyCC binned three species of *Escherichia coli* str. K-12 substr. MG1655, *Salmonella bongori* NCTC 12419 and *Salmonella enterica* subsp. arizonae serovar 62 into two clusters. It binned the contigs of *E. coli* MG1655 (99.33% recall) and *S. bongori* NCTC 12419 (66.73% recall) into the Cluster.20 (65.26% precision), while the contigs of *S. enterica* subsp. serovar 62 (85.51% recall) and the rest of *S. bongori* NCTC 12419 into the Cluster.24 (86.06% precision). It is worth noting that based on the low numbers of marker genes recorded in Cluster.summay for these two clusters (7 and 12 for Cluster.20 and Cluster.24,

respectively) and their positions located in the center of the plot, MyCC indeed provided the evidence of confidence in binning.

<u>Dataset: 64 Genomes</u>

A RayMeta assembly: Ray.contigs.fasta

A filtered assembly: 64s.fasta (contigs>=1000 bp, Header without space)

Binning gold standard: 64s.spe.txt

A coverage file: 64s.depth.txt

wget http://sourceforge.net/projects/sb2nhri/files/MyCC/Data/64s.zip

unzip 64s.zip

cd 64s

MyCC.py 64s.fasta 56mer -a 64s.depth.txt

```
manager@sb:~/Run/64s$ MyCC.py 64s.fasta 56mer -a 64s.depth.txt
20151207_1044
56mer
1_rename.py /home/manager/Run/64s/64s.fasta 1000
Seqs >= 1000 : 23602
Minimum contig lengh for first stage clustering: 1493
run Prodigal.
/opt/prodigal.linux -i My.fa -a gene.aa -d gene.nuc -f gbk -o output -s potentia
l_genes.txt
run fetchMG.
run UCLUST.
Get Feature.
2_GetFeatures_5mer_p6mer.py for fisrt stage clustering
2_GetFeatures_TimeSeries.py /home/manager/Run/64s/64s.depth.txt
2_GetFeatures_4mer.py for second stage clustering
2_GetFeatures_TimeSeries.py /home/manager/Run/64s/64s.depth.txt
3_GetMatrix.py 1493 for fisrt stage clustering
 16574 contigs entering first stage clustering
Clustering...
1_bhsne.py 20
2_ap.py /opt/ap 500 0
Cluster Correction.
to Split and Merge.
1_ClusterCorrection_Split.py 40 2
2_ClusterCorrection_Merge.py 40
Get contig by cluster.
20151207_1136
```

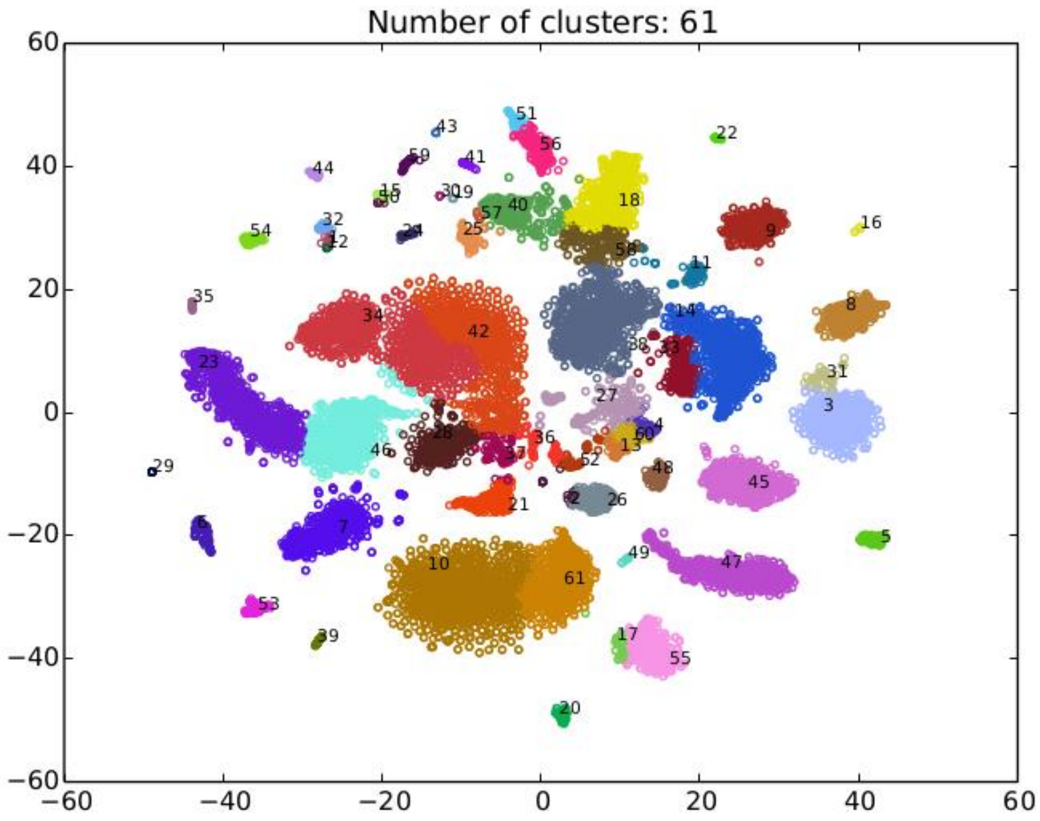

cd 20151207_1044_56mer_0.7_cov

Evaluate.py ../64s.spe.txt ../64s.fasta fasta -plot

```
manager@sb:~/Run/64s/20151207_1044_56mer_0.7_cov$ Evaluate.py ../64s.spe.txt ../
64s.fasta fasta -plot
No. of reference genomes: 64
No. of bins in evaluation: 61
No. of sequences assigned reference: 22606
No. of binned sequences: 23602
Precision: 0.731620, 0.863816
Sensitivity: 0.786207, 0.916936
```

MyReference.pdf:

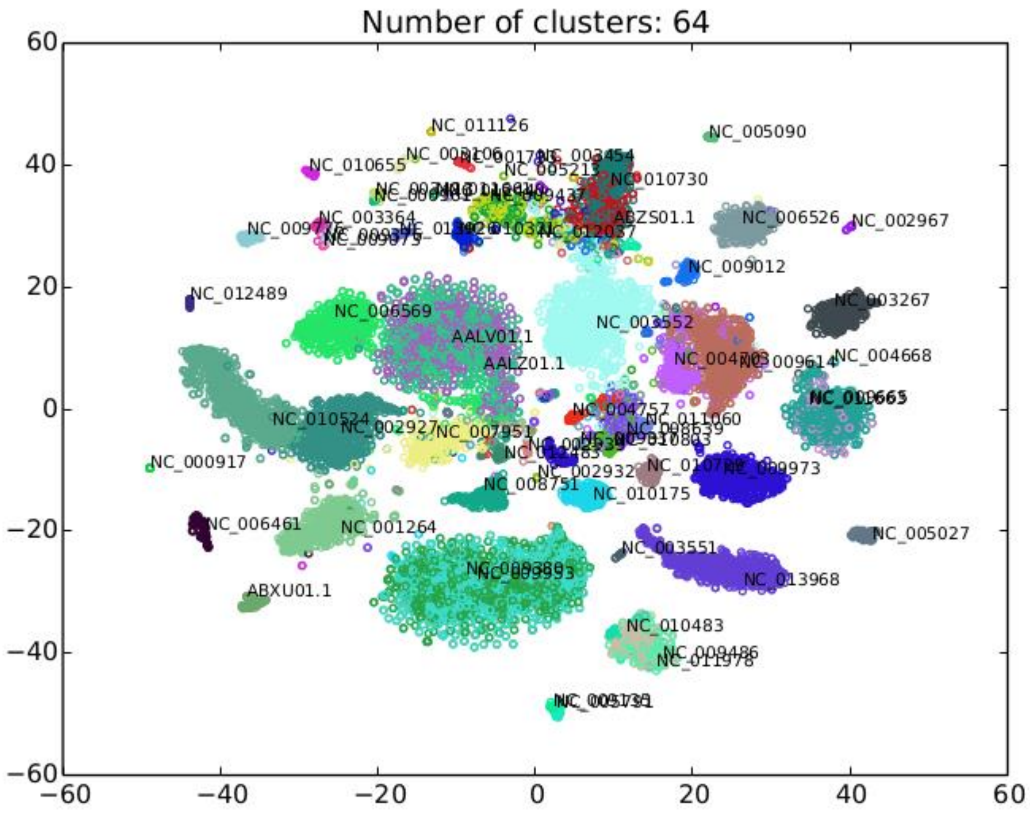

Dataset: Sharon's Dataset

Original download site: http://ggkbase.berkeley.edu/carrol/
An assembly: carrol.contigs.fa
Binning gold standard: carrol.scaffolds_to_bin.tsv

We modify the files of carrol.contigs.fa and carrol.scaffolds_to_bin.tsv to carrol.fasta (contigs>=1000 bp, Header without space) and carrol.scaffolds_to_bin.txt, respectively, for MyCC.

wget http://sourceforge.net/projects/sb2nhri/files/MyCC/Data/Sharon.zip
unzip Sharon.zip
cd Sharon
MyCC.py carrol.fasta -a My.depth.txt

```
manager@sb:~/Run/Sharon$ MyCC.py carrol.fasta -a My.depth.txt
20151207_1149
4mer
1_rename.py /home/manager/Run/Sharon/carrol.fasta 1000
Seqs >= 1000 : 2294
Minimum contig lengh for first stage clustering: 1250
run Prodigal.
/opt/prodigal.linux -i My.fa -a gene.aa -d gene.nuc -f gbk -o output -s potentia
l_genes.txt
run fetchMG.
run UCLUST.
Get Feature.
2_GetFeatures_4mer.py for fisrt stage clustering
2_GetFeatures_TimeSeries.py /home/manager/Run/Sharon/My.depth.txt
2_GetFeatures_4mer.py for second stage clustering
2_GetFeatures_TimeSeries.py /home/manager/Run/Sharon/My.depth.txt
3_GetMatrix.py 1250 for fisrt stage clustering
 1607 contigs entering first stage clustering
Clustering...
1_bhsne.py 20
2_ap.py /opt/ap 500 0
Cluster Correction.
to Split and Merge.
1_ClusterCorrection_Split.py 40 2
2_ClusterCorrection_Merge.py 40
Get contig by cluster.
20151207_1154
```

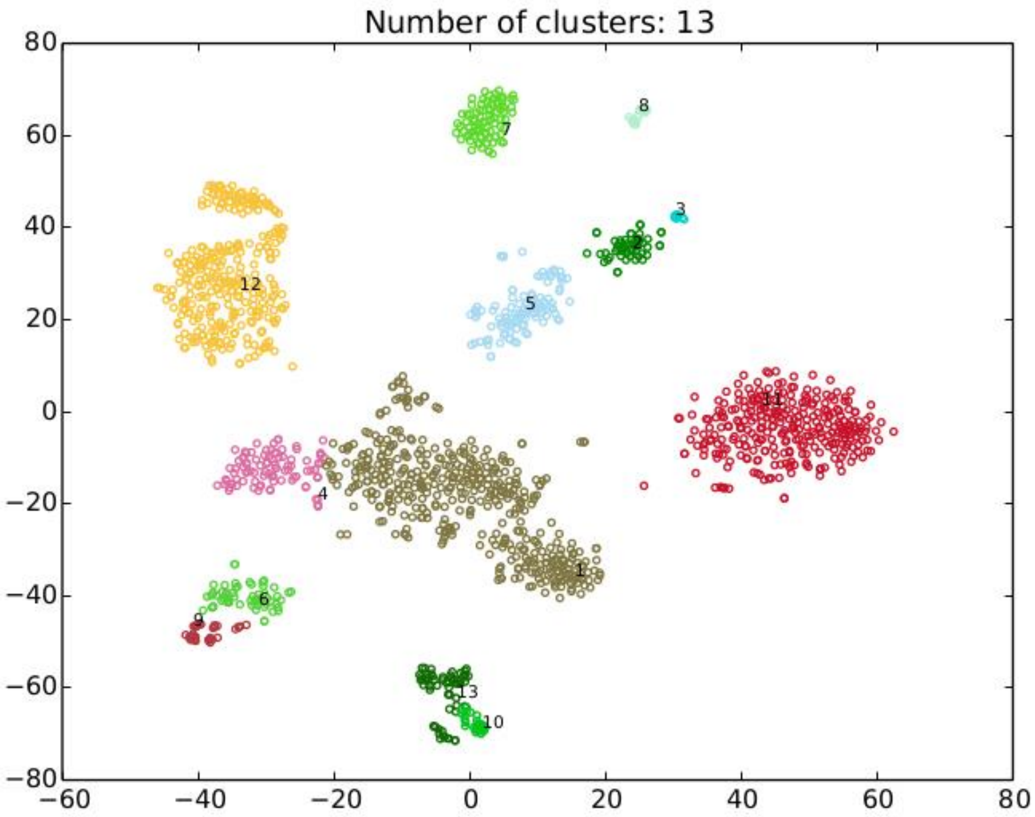

cd 20151207_1149_4mer_0.7_cov

Evaluate.py ../carrol.scaffolds_to_bin.txt ../carrol.fasta fasta -plot

```
manager@sb:~/Run/Sharon/20151207_1149_4mer_0.7_cov$ Evaluate.py ../carrol.scaffolds_to_bin.txt ../carrol.fasta fasta -plot
No. of reference genomes: 32
No. of bins in evaluation: 13
No. of sequences assigned reference: 2294
No. of binned sequences: 2294
Precision: 0.767219, 0.865632
Sensitivity: 0.908021, 0.965512
```

MyReference.pdf:

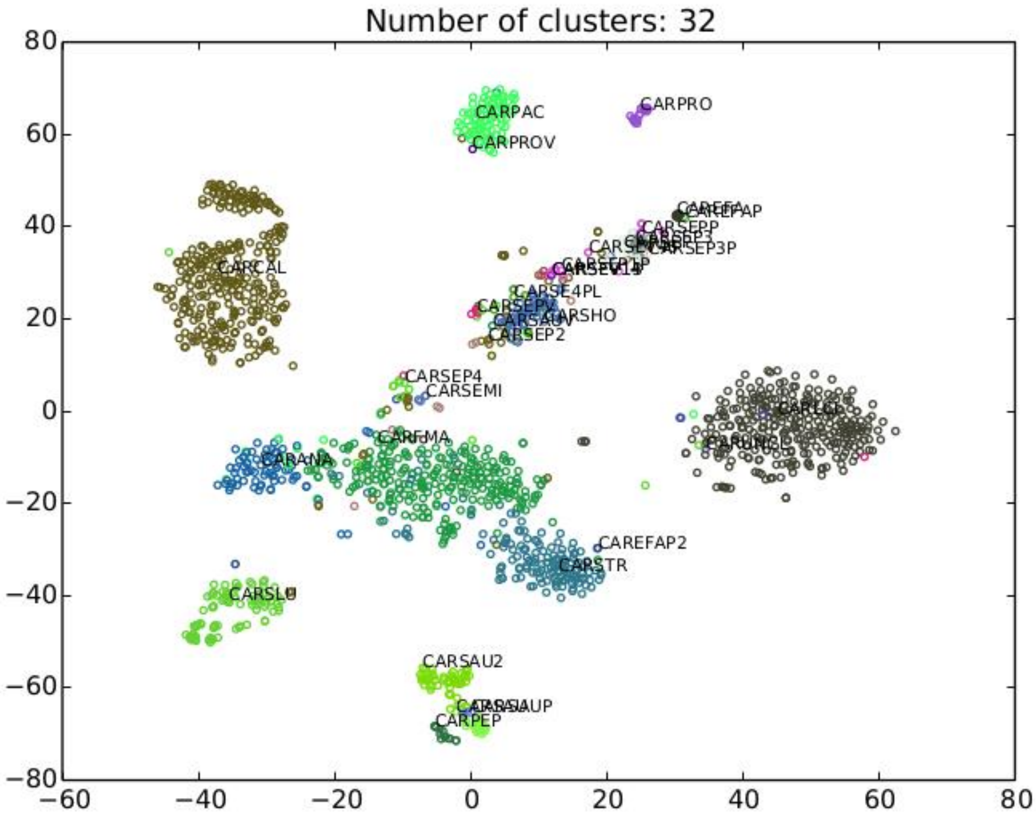

According to the description in carrol.organism_info.tsv, we found that the authors divided plasmids and viruses from a strain. Here, we group plasmids and virus into the strain and ignore some viruses.

As shown in GroupName.xls:

Name	GroupName	name	co
CARSLU	CARSLU	Staphylococcus lugdunensis	ca
CARPEP	CARPEP	Peptoniphilus sp. Carrol	ca
CARSHO	CARSHO	Staphylococcus hominis	ca
CARSAUV	CARSAU	Staphylococcus aureus virus	ca
CARSAU2	CARSAU	Staphylococcus aureus strain 2	ca
CARSAUP	CARSAU	Staphylococcus aureus plasmid	ca
CARSAU	CARSAU	Staphylococcus aureus	ca
CARPRO	CARPRO	Propionibacterium sp.	ca
CARPROV	CARPRO	Propionibacterium sp. virus	ca
CARSEP	CARSEP	Staphylococcus epidermidis strain 1	ca
CARSEPP	CARSEP	Staphylococcus epidermidis plasmid	ca
CARSEPV	CARSEP	Staphylococcus epidermidis viruses	ca
CARSEP2	CARSEP	Staphylococcus epidermidis misc.	ca
CARSEP3	CARSEP3	Staphylococcus epidermidis strain 3	ca
CARSEP4	CARSEP4	Staphylococcus epidermidis strain 4	ca
CAREFA	CAREFA	Enterococcus faecalis	ca
CAREFAP	CAREFA	Enterococcus faecalis plasmid	ca
CAREFAP2	CAREFA	Enterococcus faecalis plasmid 2	ca
CARUNCL		Carrol unclustered	ca
CARSEV46		Staphylococcus epidermidis virus 4-6	ca
CARSEV13		Staphylococcus epidermidis virus 013	ca
CARSEV14		Staphylococcus epidermidis virus 014	ca
CARSEMI		Staphylococcus epidermidis misc	ca
CARSEP1P	CARSEP	Staphylococcus epidermidis strain 1 plasmids	ca
CARSEP3P	CARSEP3	Staphylococcus epidermidis strain 3 plasmids	ca
CARSE4PL	CARSEP4	Staphylococcus epidermidis strain 4 plasmids	ca
ACDRDN		Redundant	ca
CARCAL	CARCAL	Candida albicans	ca
CARFMA	CARFMA	Finegoldia magna	ca
CARLCI	CARLCI	Leuconostoc citreum	ca
CARANA	CARANA	Anaerococcus sp.	ca
CARPAC	CARPAC	Propionibacterium acnes	ca
CARSTR	CARSTR	Streptococcus sp.	ca

Evaluate.py ../carrol.scaffolds_to_bin.groupname.txt ../carrol.fasta fasta -plot

```
manager@sb:~/Run/Sharon/20151207_1149_4mer_0.7_cov$ Evaluate.py ../carrol.scaffolds_to_bin.groupname.txt ../carrol.fasta fasta -plot
No. of reference genomes: 15
No. of bins in evaluation: 13
No. of sequences assigned reference: 2270
No. of binned sequences: 2294
Precision: 0.782819, 0.877007
Sensitivity: 0.889427, 0.955113
```

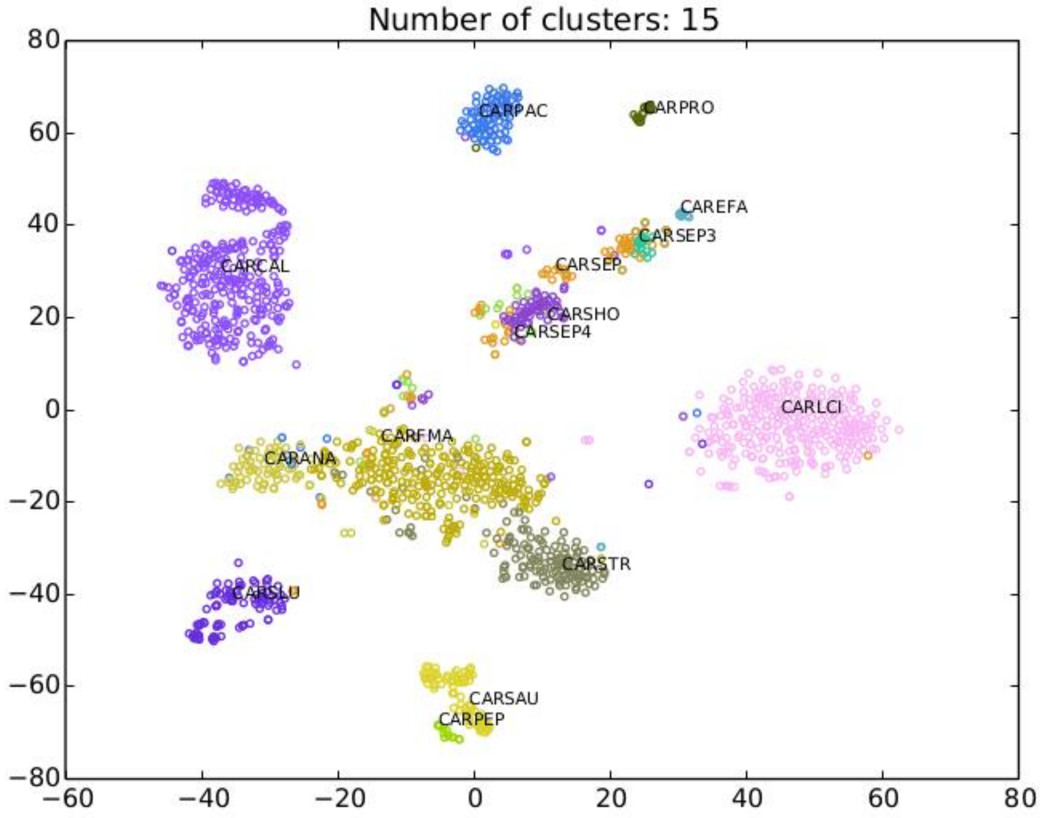

MyCC.py carrol.fasta -a My.depth.txt -keep

```
manager@sb:~/Run/Sharon$ MyCC.py carrol.fasta -a My.depth.txt -keep
20151207_1158
4mer
1_rename.py /home/manager/Run/Sharon/carrol.fasta 1000
Seqs >= 1000 : 2294
Minimum contig lengh for first stage clustering: 1250
run Prodigal.
/opt/prodigal.linux -i My.fa -a gene.aa -d gene.nuc -f gbk -o output -s potentia
l_genes.txt
run fetchMG.
run UCLUST.
Get Feature.
2_GetFeatures_4mer.py for fisrt stage clustering
2_GetFeatures_TimeSeries.py /home/manager/Run/Sharon/My.depth.txt
2_GetFeatures_4mer.py for second stage clustering
2_GetFeatures_TimeSeries.py /home/manager/Run/Sharon/My.depth.txt
3_GetMatrix.py 1250 for fisrt stage clustering
 1607 contigs entering first stage clustering
Clustering...
1_bhsne.py 20
2_ap.py /opt/ap 500 0
Cluster Correction.
to Split and Merge.
1_ClusterCorrection_Split.py 40 2
2_ClusterCorrection_Merge.py 40
Get contig by cluster.
20151207_1203
[illegible]
```

By adding the option of "-keep", MyCC keeps temporary files in the output folder:

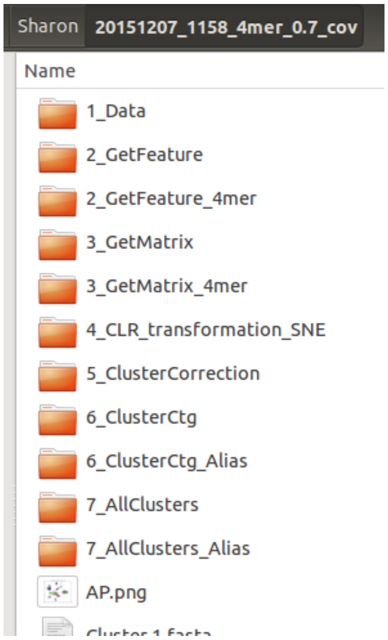

So, you can find the visualization plots including Barnes-Hut-SNT and Affinity Propagation clustering (as shown below) in the fold of 4_CLR_transformation_SNE.

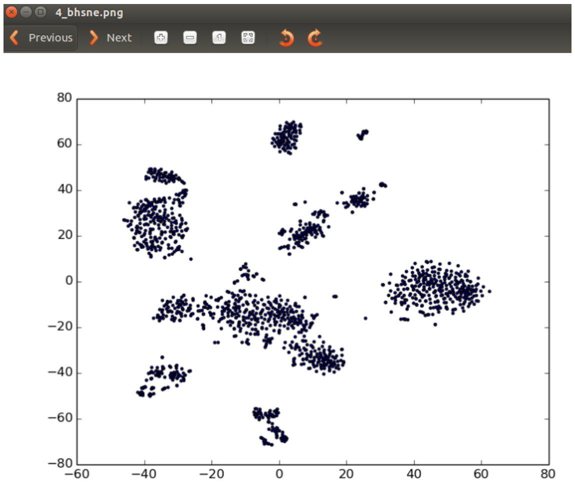

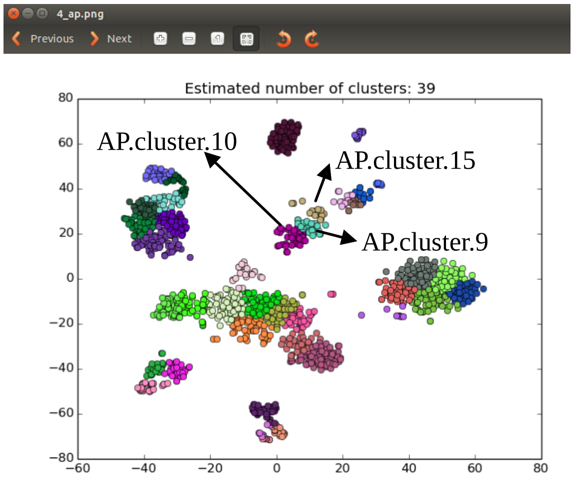

According to 20151207_1158_4mer_0.7_cov/5_ClusterCorrection/0_Cog_AP.txt,
9 COGs: COG0012, COG0016, COG18, COG0081, COG0087, COG0172, COG0185, COG0201 and COG0215 in AP.cluster.9
8 COGs: COG0052, COG0102, COG0124, COG0495, COG0525, COG0533, COG0541 and COG0552 in AP.cluster.10
0 COG in AP.cluster.15
After iterative splitting and merging processes, these three clusters were finally merged to form a corrected cluster (*e.g.*, Cluster.5 in page 31).

cd 20151207_1158_4mer_0.7_cov/

Evaluate.py ../carrol.scaffolds_to_bin.txt ../carrol.fasta fasta -plot

```
manager@sb:~/Run/Sharon/20151207_1158_4mer_0.7_cov$ Evaluate.py ../carrol.scaffo
lds_to_bin.txt ../carrol.fasta fasta -plot
No. of reference genomes: 32
No. of bins in evaluation: 13
No. of sequences assigned reference: 2294
No. of binned sequences: 2294
Precision: 0.767219, 0.865632
Sensitivity: 0.908021, 0.965512
```

You are able to evaluate the binning results in the first stage: (contig length >= the length of the 1606th contig (2294*0.7) entering the first stage)

cd 6_ClusterCtg_Alias

Evaluate.py ../../carrol.scaffolds_to_bin.txt ../../carrol.fasta fasta

```
manager@sb:~/Run/Sharon/20151207_1158_4mer_0.7_cov/6_ClusterCtg_Alias$ Evaluate.
py ../../carrol.scaffolds_to_bin.txt ../../carrol.fasta fasta
No. of reference genomes: 32
No. of bins in evaluation: 13
No. of sequences assigned reference: 2294
No. of binned sequences: 1607
Precision: 0.792782, 0.870529
Sensitivity: 0.644289, 0.941130
```

By comparing the binning result with the binning gold standard, we found that the sequences in Cluster.3 represent the genome of *Enterococcus faecalis*, which was reconstructed from infant gut microbiome by Sharon *et al*., not from the so-called "reference genomes".

The precision of Cluster.3: 96.98%

The recall of *Enterococcus faecalis*: 100%

```
Cluster.3.fasta:0.969841(2877608/2967093)      CAREFA:2877608  CAREFAP:88476   CARSEP2:1009
CAREFA:1.000000(2877608/2877608)       Cluster.3.fasta:2877608
```

Dataset: Drosophila dataset

A RayMeta assembly: my.ctg.fa

A coverage file: My.depth.txt

Cluster.1.COGs.fasta

Cluster.5.COGs.fasta

Cluster.6.COGs.fasta

Cluster.5.faa

Cluster.5.tbl

Cluster.6.faa

Cluster.6.tbl

wget http://sourceforge.net/projects/sb2nhri/files/MyCC/Data/fly.zip

unzip fly.zip

cd fly

MyCC.py my.ctg.fa -a My.depth.txt -keep

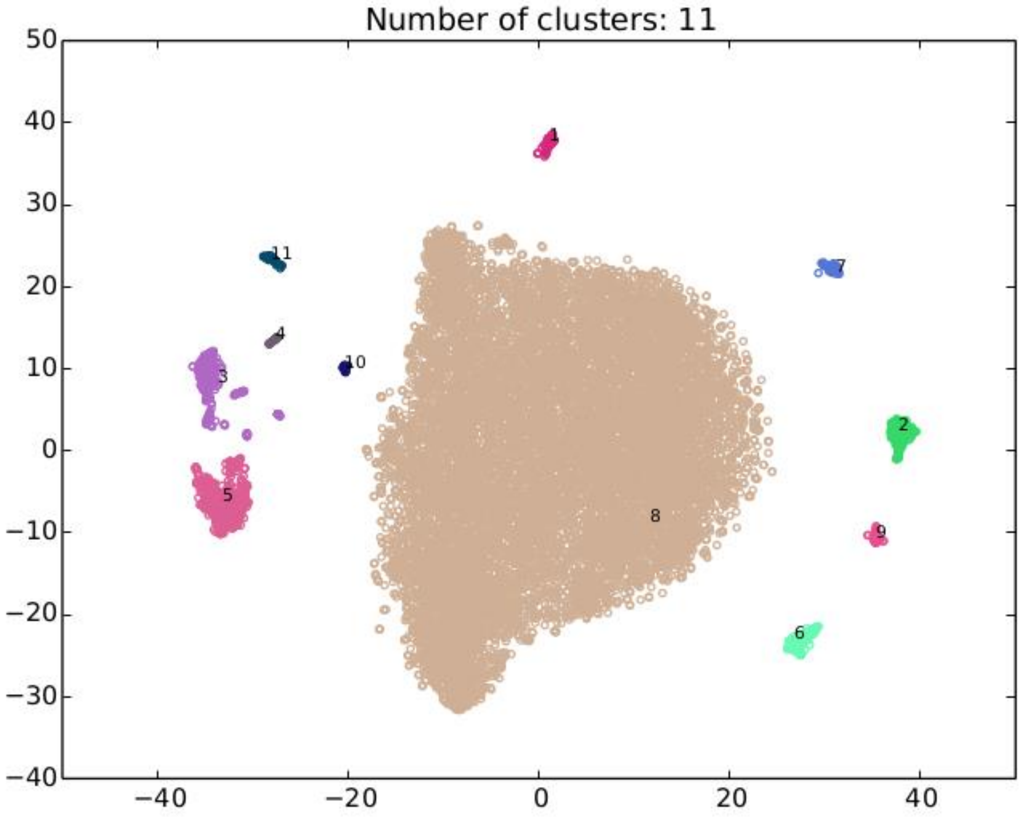

Cluster.summary

```
Cluster WholeGenome      N50     NoOfCtg LongestCtgLen   AvgLenOfCtg     Cogs
Cluster.1.fasta 1412423 22190   110     54381   6542    39
Cluster.2.fasta 1418844 6729    274     24492   2595    10
Cluster.3.fasta 3996074 15023   465     55244   4297    27
Cluster.4.fasta 224814  5214    50      12319   2309    4
Cluster.5.fasta 2554723 3661    828     20009   1545    31
Cluster.6.fasta 1203054 5915    291     26593   2075    33
Cluster.7.fasta 452006  2227    220     8247    1032    7
Cluster.8.fasta 108511839       9586    19446   100844  2790    14
Cluster.9.fasta 324829  3159    119     7328    1366    5
Cluster.10.fasta        130137  3779    48      15046   1381    0
Cluster.11.fasta        397064  3684    134     10352   1499    9
```

The binning results were assessed by CheckM to produce a summary table:

Bin ID	Marker lineage	Completeness	Contamination	Strain heterogeneity
Cluster.1	c__Gammaproteobacteria (UID4387)	93.22	0	0
Cluster.5	o__Rhodospirillales (UID3754)	90.63	4.79	53.85
Cluster.3	o__Rhodospirillales (UID3754)	90.24	2.9	25
Cluster.6	o__Lactobacillales (UID463)	89.56	1.47	75
Cluster.8	k__Archaea (UID2)	75.63	44.89	2.42
Cluster.2	o__Lactobacillales (UID463)	37.57	0.16	0
Cluster.7	k__Bacteria (UID203)	28.92	0	0
Cluster.9	k__Bacteria (UID203)	21.87	1.15	100
Cluster.11	o__Lactobacillales (UID463)	11.08	0	0
Cluster.4	o__Rhodospirillales (UID3754)	7.47	0	0
Cluster.10	root (UID1)	0	0	0

This table shows that the upper four clusters (Cluster.1, Cluster.3, Cluster. 5 and Cluster.6) are of good quality: near complete and with low contamination.

Among the 11 clusters, three clusters (Cluster.1, Cluster. 5 and Cluster.6) have large marker gene counts (39, 31 and 33, respectively) in MyCC’s summary. We further examined these three clusters.

For example, looking into Cluster.6.fasta
Blast the longest contig (contig-2364000000) against Nucleotide collection:

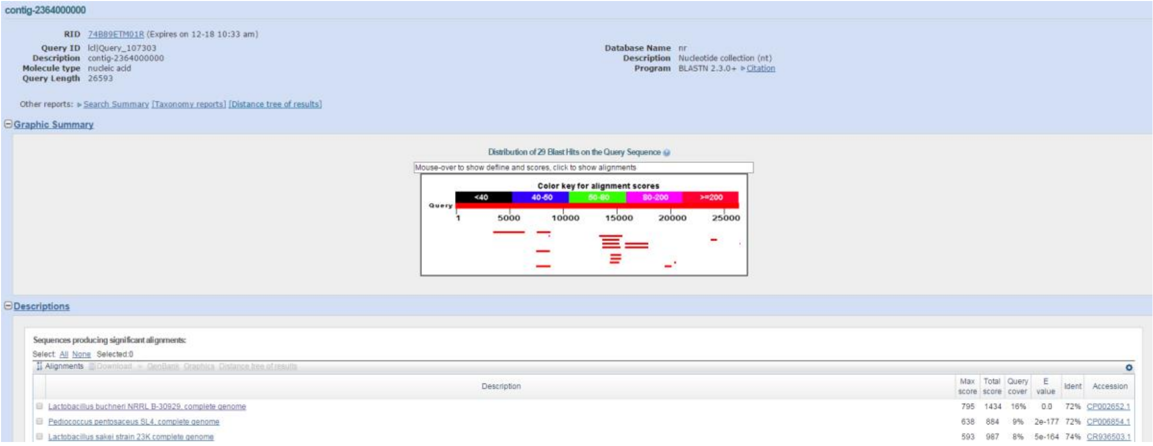

Blast contig-54919000000 against Nucleotide collection:

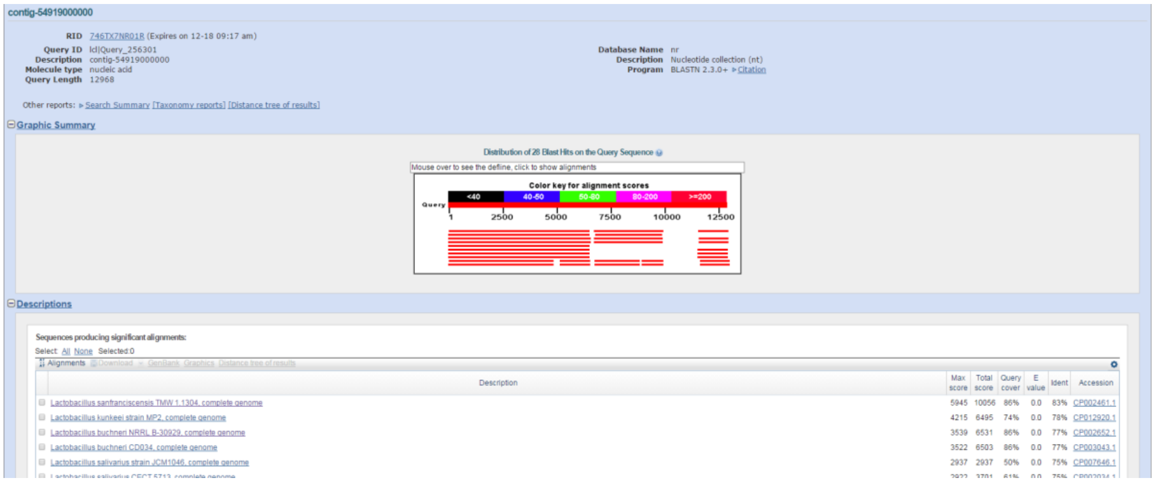

……..

The above results suggst that sequences in this cluster are differenct from the nucleotide collection.

With Alias.txt (in the folder of 1_Data), we know the contig “contig-5491900000” is Seq_21793_len_12968.

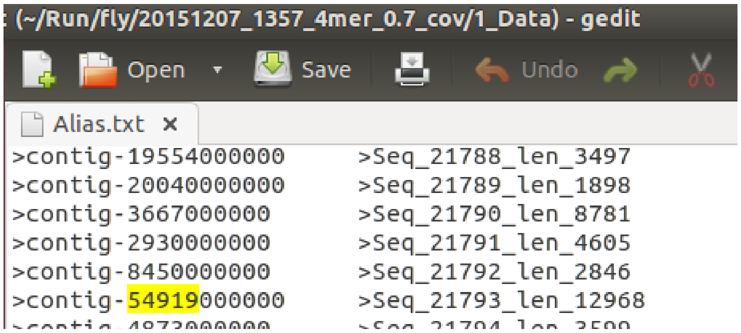

Looking into files of marker gene (*e.g.*, COG0093.faa in 1_Data/output), protein sequences of marker genes can be retrieved.

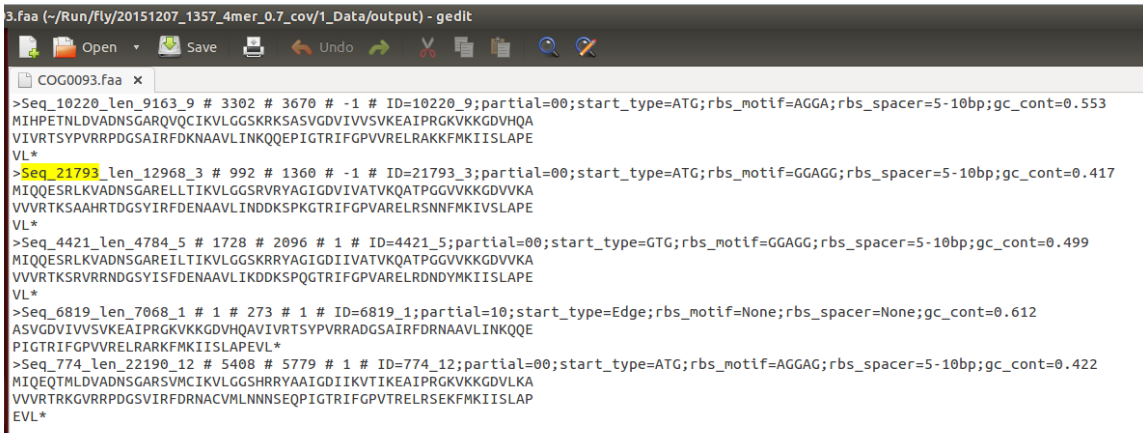

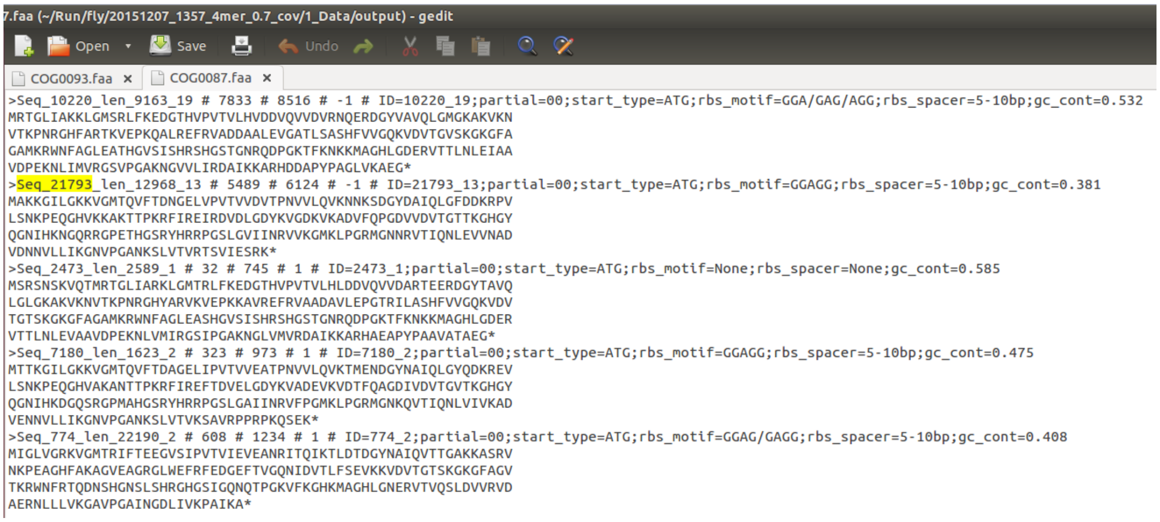

```
7.faa (~/Run/fly/20151207_1357_4mer_0.7_cov/1_Data/output) - gedit
Open  Save  Undo
COG0093.faa ×  COG0087.faa ×
>Seq_10220_len_9163_19 # 7833 # 8516 # -1 # ID=10220_19;partial=00;start_type=ATG;rbs_motif=GGA/GAG/AGG;rbs_spacer=5-10bp;gc_cont=0.532
MRTGLIAKKLGMSRLFKEDGTHVPVTVLHVDDVQVVDVRNQERDGYVAVQLGMGKAKVKN
VTKPNRGHFARTKVEPKQALREFRVADDAALEVGATLSASHFVVGQKVDVTGVSKGKGFA
GAMKRWNFAGLEATHGVSISHRSHGSTGNRQDPGKTFKNKKMAGHLGDERVTTLNLEIAA
VDPEKNLIMVRGSVPGAKNGVVLIRDAIKKARHDDAPYPAGLVKAEG*
>Seq_21793_len_12968_13 # 5489 # 6124 # -1 # ID=21793_13;partial=00;start_type=ATG;rbs_motif=GGAGG;rbs_spacer=5-10bp;gc_cont=0.381
MAKKGILGKKVGMTQVFTDNGELVPVTVVDVTPNVVLQVKNNKSDGYDAIQLGFDDKRPV
LSNKPEQGHVKKAKTTPKRFIREIRDVDLGDYKVGDKVKADVFQPGDVVDVTGTTKGHGY
QGNIHKNGQRRGPETHGSRYHRRPGSLGVIINRVVKGMKLPGRMGNNRVTIQNLEVVNAD
VDNNVLLIKGNVPGANKSLVTVRTSVIESRK*
>Seq_2473_len_2589_1 # 32 # 745 # 1 # ID=2473_1;partial=00;start_type=ATG;rbs_motif=None;rbs_spacer=None;gc_cont=0.585
MSRSNSKVQTMRTGLIARKLGMTRLFKEDGTHVPVTVLHLDDVQVVDARTEERDGYTAVQ
LGLGKAKVKNVTKPNRGHYARVKVEPKKAVREFRVAADAVLEPGTRILASHFVVGQKVDV
TGTSKGKGFAGAMKRWNFAGLEASHGVSISHRSHGSTGNRQDPGKTFKNKKMAGHLGDER
VTTLNLEVAAVDPEKNLVMIRGSIPGAKNGLVMVRDAIKKARHAEAPYPAAVATAEG*
>Seq_7180_len_1623_2 # 323 # 973 # 1 # ID=7180_2;partial=00;start_type=ATG;rbs_motif=GGAGG;rbs_spacer=5-10bp;gc_cont=0.475
MTTKGILGKKVGMTQVFTDAGELIPVTVVEATPNVVLQVKTMENDGYNAIQLGYQDKREV
LSNKPEQGHVAKANTTPKRFIREFTDVELGDYKVADEVKVDTFQAGDIVDVTGVTKGHGY
QGNIHKDGQSRGPMAHGSRYHRRPGSLGAIINRVFPGMKLPGRMGNKQVTIQNLVIVKAD
VENNVLLIKGNVPGANKSLVTVKSAVRPPRPKQSEK*
>Seq_774_len_22190_2 # 608 # 1234 # 1 # ID=774_2;partial=00;start_type=ATG;rbs_motif=GGAG/GAGG;rbs_spacer=5-10bp;gc_cont=0.408
MIGLVGRKVGMTRIFTEEGVSIPVTVIEVEANRITQIKTLDTDGYNAIQVTTGAKKASRV
NKPEAGHFAKAGVEAGRGLWEFRFEDGEFTVGQNIDVTLFSEVKKVDVTGTSKGKGFAGV
TKRWNFRTQDNSHGNSLSHRGHGSIGQNQTPGKVFKGHKMAGHLGNERVTVQSLDVVRVD
AERNLLLVKGAVPGAINGDLIVKPAIKA*
```

Accordingly, a file containng the protein sequences of marker genes for Cluster.6 was obtained. (**Cluster.6.COGs.fasta**)

Use SmartBLAST (http://blast.ncbi.nlm.nih.gov/smartblast/):

>contig-54919000000_COG0186.faa

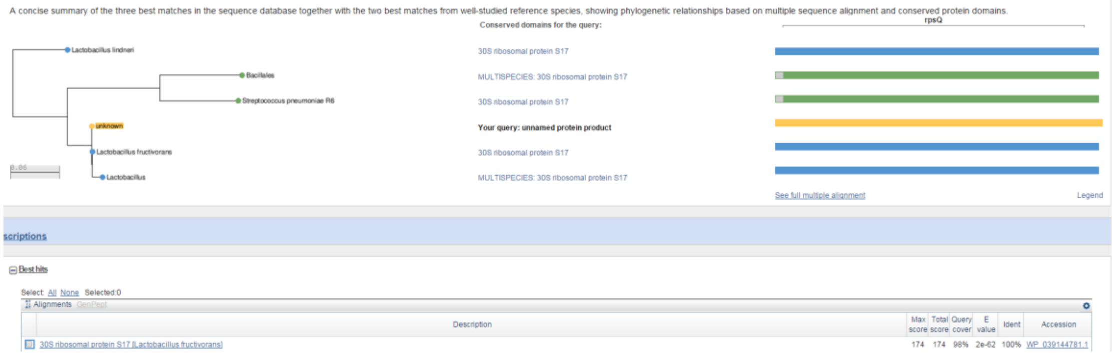

>contig-1355000000_COG0012.faa

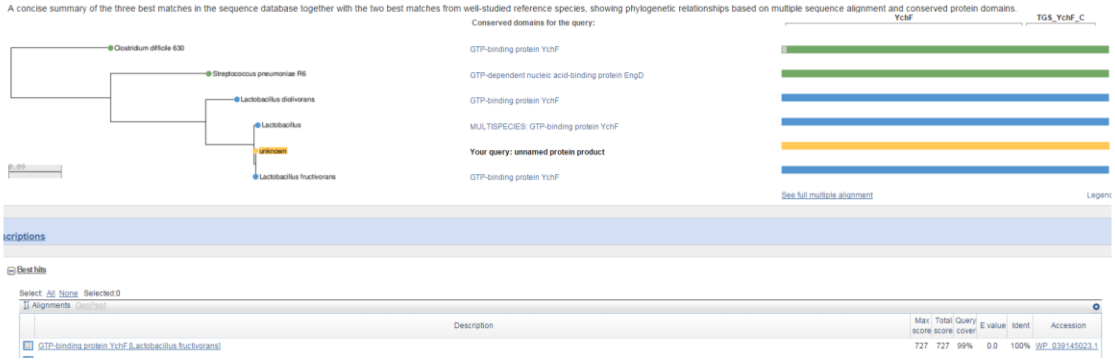

>contig-1324000000_COG0533.faa

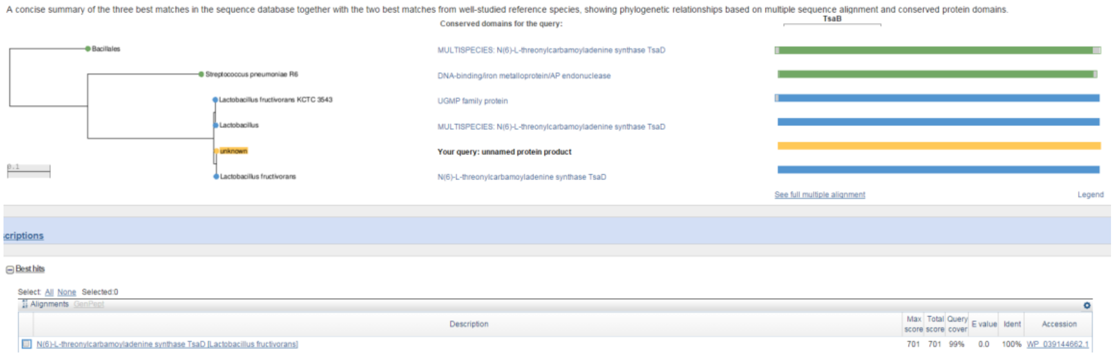

…….

The above results (28 out of the 33 COGs) suggst that the closest species to this cluster is *Lactobacillus fructivorans*.

Similarly, according to the protein sequences of marker genes for Cluster.5 (**Cluster.5.COGs.fasta**), we found the closest species to Cluster.5 is *Acetobacter pasteurianus*.

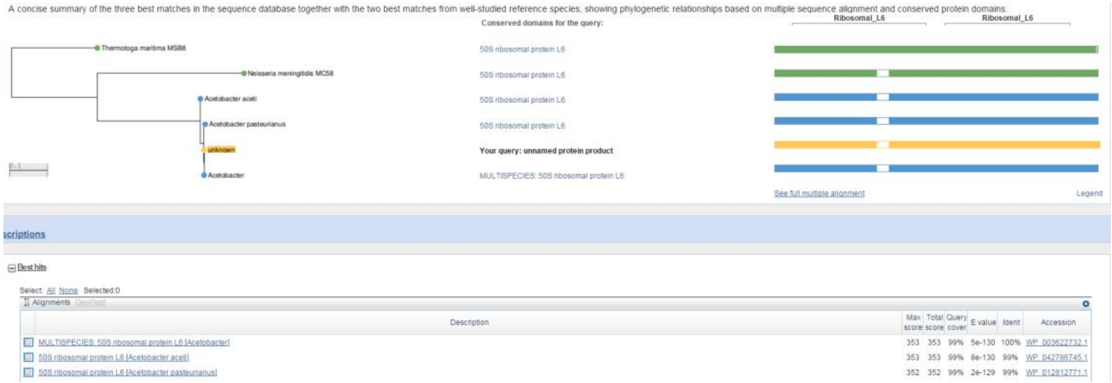

…….

However, the protein sequences of marker genes for Cluster.1 (**Cluster.1.COGs.fasta**) suggest that the sequences in this cluster may be from a new species.

>contig-55588000000_COG0124.faa

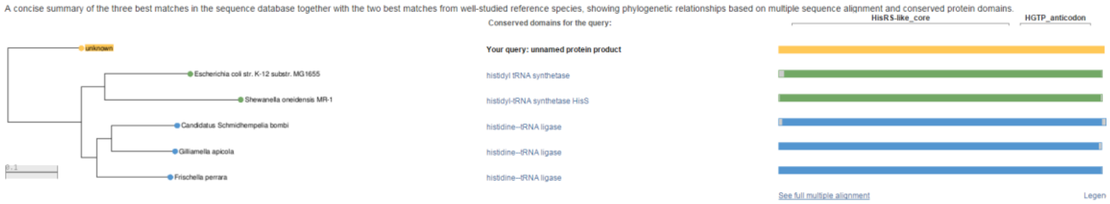

scriptions

Best hits

Select: All None Selected:0

Alignments GenPept

Description	Max score	Total score	Query cover	E value	Ident	Accession
histidine--tRNA ligase [Frischella perrara]	579	579	99%	0.0	64%	WP_039105911.1
histidine--tRNA ligase [Candidatus Schmidhempelia bombi]	573	573	99%	0.0	63%	WP_024495198.1
histidine--tRNA ligase [Gilliamella apicola]	566	566	98%	0.0	63%	WP_034913295.1
histidyl tRNA synthetase [Escherichia coli str. K-12 substr. MG1655]	538	538	98%	0.0	59%	NP_417009.1
histidyl-tRNA synthetase HisS [Shewanella oneidensis MR-1]	511	511	98%	7e-178	58%	NP_718865.1

>contig-55668000000_COG0525.faa

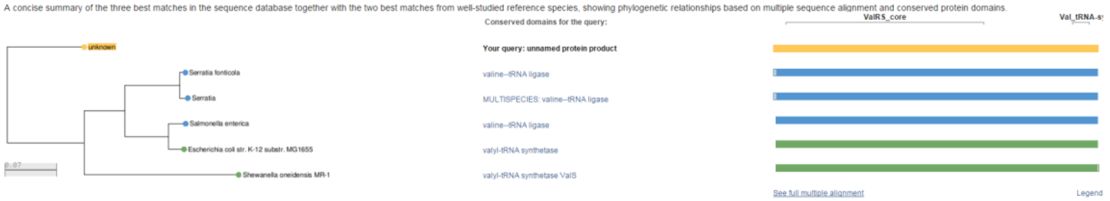

scriptions

Best hits

Select: All None Selected:0

Alignments GenPept

Description	Max score	Total score	Query cover	E value	Ident	Accession
valine--tRNA ligase [Serratia fonticola]	1461	1461	99%	0.0	71%	WP_021807812.1
valine--tRNA ligase [Salmonella enterica]	1457	1457	99%	0.0	71%	WP_016507497.1
MULTISPECIES: valine--tRNA ligase [Serratia]	1454	1454	99%	0.0	71%	WP_025122597.1
valyl-tRNA synthetase [Escherichia coli str. K-12 substr. MG1655]	1451	1451	99%	0.0	72%	NP_418679.1
valyl-tRNA synthetase ValS [Shewanella oneidensis MR-1]	1367	1367	99%	0.0	67%	NP_718977.1

>contig-22000000_COG0184.faa

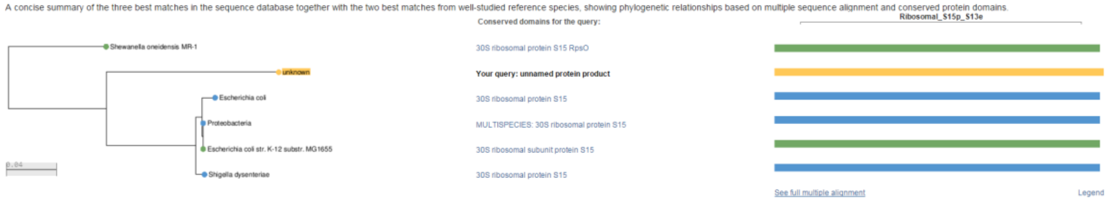

scriptions

Best hits

Select: All None Selected:0

Alignments GenPept

Description	Max score	Total score	Query cover	E value	Ident	Accession
30S ribosomal protein S15 [Escherichia coli]	150	150	98%	4e-53	81%	WP_001667872.1
30S ribosomal protein S15 [Shigella dysenteriae]	150	150	98%	7e-53	81%	WP_000059468.1
MULTISPECIES: 30S ribosomal protein S15 [Proteobacteria]	149	149	98%	8e-53	81%	WP_000059466.1

…….

The sequences of Cluster.5.fasta and Cluster.6.fasta were annotated by Prokka with high-quality reference genomes (http://www.ncbi.nlm.nih.gov/genome/browse/reference/) as genus db.

Prokka --genus Acetobacter --usegenus Cluster.5.fasta

organism: Acetobacter species strain

contigs: 828

bases: 2554723
tRNA: 22
tmRNA: 1
CDS: 2447

Prokka --genus Lactobacillus--usegenus Cluster.6.fasta
organism: Lactobacillus species strain
contigs: 291
bases: 1203054
tRNA: 12
CDS: 1055

FASTA files of translated coding genes (**Cluster.5.faa and Cluster.6.faa**) and feature tables (**Cluster.5.tbl, and Cluster.6.tbl**) are provided in the fly.zip.

Docker of MyCC

Docker in Ubuntu (*This test was performed in VirtualBox of MyCC*)

curl -sSL https://get.docker.com/ | sudo sh

(Password for manager: manager)

Start docker:

```
manager@sb:~$ sudo service docker start
start: Job is already running: docker
```

Check status:

sudo service docker status

```
manager@sb:~$ sudo service docker status
docker start/running, process 6798
```

Pull the docker image of MyCC:

sudo docker run -t -i -v /home/manager/Run:/Run 990210oliver/mycc.docker:v1 /bin/bash

```
manager@sb:~$ sudo docker run -t -i -v /home/manager/Run:/Run 990210oliver/mycc.
docker:v1 /bin/bash
Unable to find image '990210oliver/mycc.docker:v1' locally
v1: Pulling from 990210oliver/mycc.docker
2880a3395ede: Pull complete
515565c29c94: Pull complete
98b15185dba7: Pull complete
2ce633e3e9c9: Pull complete
9d4ef3367e93: Pull complete
d242658d2723: Pull complete
a2cbf632e138: Pull complete
b2b78f236713: Pull complete
ecc0cd64d372: Pull complete
130b2aedb075: Pull complete
7fb33e8f384f: Pull complete
8dd940730acf: Pull complete
6b596cda3368: Pull complete
66158a4b5885: Pull complete
c54a1a4bc7d7: Pull complete
b7c3f11e4e9a: Pull complete
0fd69001d6b6: Pull complete
Digest: sha256:3832de4d8f16c0fa097a9c01dc7a51b21acab8da56d2ef453ab822ad415ebac6
Status: Downloaded newer image for 990210oliver/mycc.docker:v1
```

cd Run/10s

MyCC.py 10s.fasta

```
root@f8fc39cf5ad3:/Run/10s# MyCC.py 10s.fasta
20151207_0424
4mer
1_rename.py /Run/10s/10s.fasta 1000
Seqs >= 1000 : 2185
Minimum contig lengh for first stage clustering: 3786
run Prodigal.
/opt/prodigal.linux -i My.fa -a gene.aa -d gene.nuc -f gbk -o output -s potentia
l_genes.txt
run fetchMG.
run UCLUST.
Get Feature.
2_GetFeatures_4mer.py for fisrt stage clustering
2_GetFeatures_4mer.py for second stage clustering
3_GetMatrix.py 3786 for fisrt stage clustering
 1538 contigs entering first stage clustering
Clustering...
1_bhsne.py 20
2_ap.py /opt/ap 500 0
Cluster Correction.
to Split and Merge.
1_ClusterCorrection_Split.py 40 2
2_ClusterCorrection_Merge.py 40
Get contig by cluster.
20151207_0429
```

cd 20151207_0424_4mer_0.7/

Evaluate.py ../10s.spe.txt ../10s.fasta fasta -plot -split

```
root@f8fc39cf5ad3:/Run/10s/20151207_0424_4mer_0.7# Evaluate.py ../10s.spe.txt ..
/10s.fasta fasta -plot -split
No. of reference genomes: 10
No. of bins in evaluation: 10
No. of sequences assigned reference: 2172
No. of binned sequences: 2185
Precision: 0.944291, 0.974709
Sensitivity: 0.944291, 0.974709
```

exit

Run MyCC:

sudo docker run -t -i -v /home/manager/Run:/Run 990210oliver/mycc.docker:v1 /bin/bash

Supplementary Methods

Evaluating binning performance by benchmark.R (http://portal.nersc.gov/dna/RD/Metagenome_RD/MetaBAT/Files/):

```
wget http://sourceforge.net/projects/sb2nhri/files/MyCC/Data/benchmark/Sharon.zip
unzip Sharon.zip -d Sharon
R
---In R console---
source('http://portal.nersc.gov/dna/RD/Metagenome_RD/MetaBAT/Files/benchmark.R')
setwd('yourpath/Sharon/')
res <- list(MyCC=calcPerf('MaxBin','MyCC'),ConCOCT=calcPerf('MaxBin','ConCOCT'),
MaxBin=calcPerf('MaxBin','maxbin'), MetaBAT=calcPerf('MaxBin','MetaBat'),
GroopM=calcPerf('MaxBin','GroopM'))
plotPerf(res,xlim=50)
pdf('infant.pdf',width=8,height=8)
plotPerf(res,xlim=50)
dev.off()
```
